# Human snoRNA-93 is processed into a microRNA-like RNA that promotes breast cancer cell invasion

**DOI:** 10.1038/s41523-017-0032-8

**Published:** 2017-07-10

**Authors:** Dillon G. Patterson, Justin T. Roberts, Valeria M. King, Dominika Houserova, Emmaline C. Barnhill, Aline Crucello, Caroline J. Polska, Lucas W. Brantley, Garrett C. Kaufman, Michael Nguyen, Megann W. Santana, Ian A. Schiller, Julius S. Spicciani, Anastasia K. Zapata, Molly M. Miller, Timothy D. Sherman, Ruixia Ma, Hongyou Zhao, Ritu Arora, Alexander B. Coley, Melody M. Zeidan, Ming Tan, Yaguang Xi, Glen M. Borchert

**Affiliations:** 1 Department of Biology, University of South Alabama, Mobile, AL 36688 USA; 2 Department of Pharmacology, USA College of Medicine, Mobile, AL 36688 USA; 3 Department of Genetics, LSUHSC, New Orleans, LA 70112 USA; 4 Stanley S. Scott Cancer Center, LSUHSC, New Orleans, LA 70112 USA; 5 Center for Cell Death and Metabolism, Mitchell Cancer Institute, University of South Alabama, Mobile, AL 36604 USA; 6 Department of Biochemistry and Molecular Biology, USA College of Medicine, Mobile, AL 36688 USA; 7 Department of Microbiology and Immunology, Emory University School of Medicine, Atlanta, GA 30322 USA; 8 Department of Biochemistry and Molecular Genetics, University of Colorado School of Medicine, Aurora, CO 80045 USA

## Abstract

Genetic searches for tumor suppressors have recently linked small nucleolar RNA misregulations with tumorigenesis. In addition to their classically defined functions, several small nucleolar RNAs are now known to be processed into short microRNA-like fragments called small nucleolar RNA-derived RNAs. To determine if any small nucleolar RNA-derived RNAs contribute to breast malignancy, we recently performed a RNA-seq-based comparison of the small nucleolar RNA-derived RNAs of two breast cancer cell lines (MCF-7 and MDA-MB-231) and identified small nucleolar RNA-derived RNAs derived from 13 small nucleolar RNAs overexpressed in MDA-MB-231s. Importantly, we find that inhibiting the most differentially expressed of these small nucleolar RNA-derived RNAs (sdRNA-93) in MDA-MB-231 cells results primarily in a loss of invasiveness, whereas increased sdRNA-93 expression in either cell line conversely results in strikingly enhanced invasion. Excitingly, we recently determined sdRNA-93 expressions in small RNA-seq data corresponding to 116 patient tumors and normal breast controls, and while we find little sdRNA-93 expression in any of the controls and only sporadic expression in most subtypes, we find robust expression of sdRNA-93 in 92.8% of Luminal B Her2+tumors. Of note, our analyses also indicate that at least one of sdRNA-93’s endogenous roles is to regulate the expression of Pipox, a sarcosine metabolism-related protein whose expression significantly correlates with distinct molecular subtypes of breast cancer. We find sdRNA-93 can regulate the Pipox 3′UTR via standard reporter assays and that manipulating endogenous sdRNA-93 levels inversely correlates with altered Pipox expression. In summary, our results strongly indicate that sdRNA-93 expression actively contributes to the malignant phenotype of breast cancer through participating in microRNA-like regulation.

## RNA: Small nucleolar-derived RNA contributes to tumor invasiveness

A short microRNA-like fragment excised from a small nucleolar RNA (called a snoRNA-derived RNA or sdRNA) contributes to the invasiveness of breast cancer cells. Glen Borchert from the University of South Alabama, USA, and colleagues compared the expression of sdRNAs between a primary breast cancer cell line and a metastatic one. They identified 13 sdRNAs with markedly different abundances. Blocking the sdRNA with the biggest expression differential sdRNA-93-decreased cellular invasion, whereas increasing its abundance enhanced tumor cell invasiveness. Looking at human tissues, sdRNA-93 was routinely expressed in biopsies taken from patients with the luminal HER2-positive form of breast cancer, less often in other tumor types and almost never in healthy breast samples. Hinting at its function, the researchers showed that sdRNA-93 targets and regulates a gene contributing to specific molecular subtypes of breast cancer.

## Introduction

**Fig. 1:**
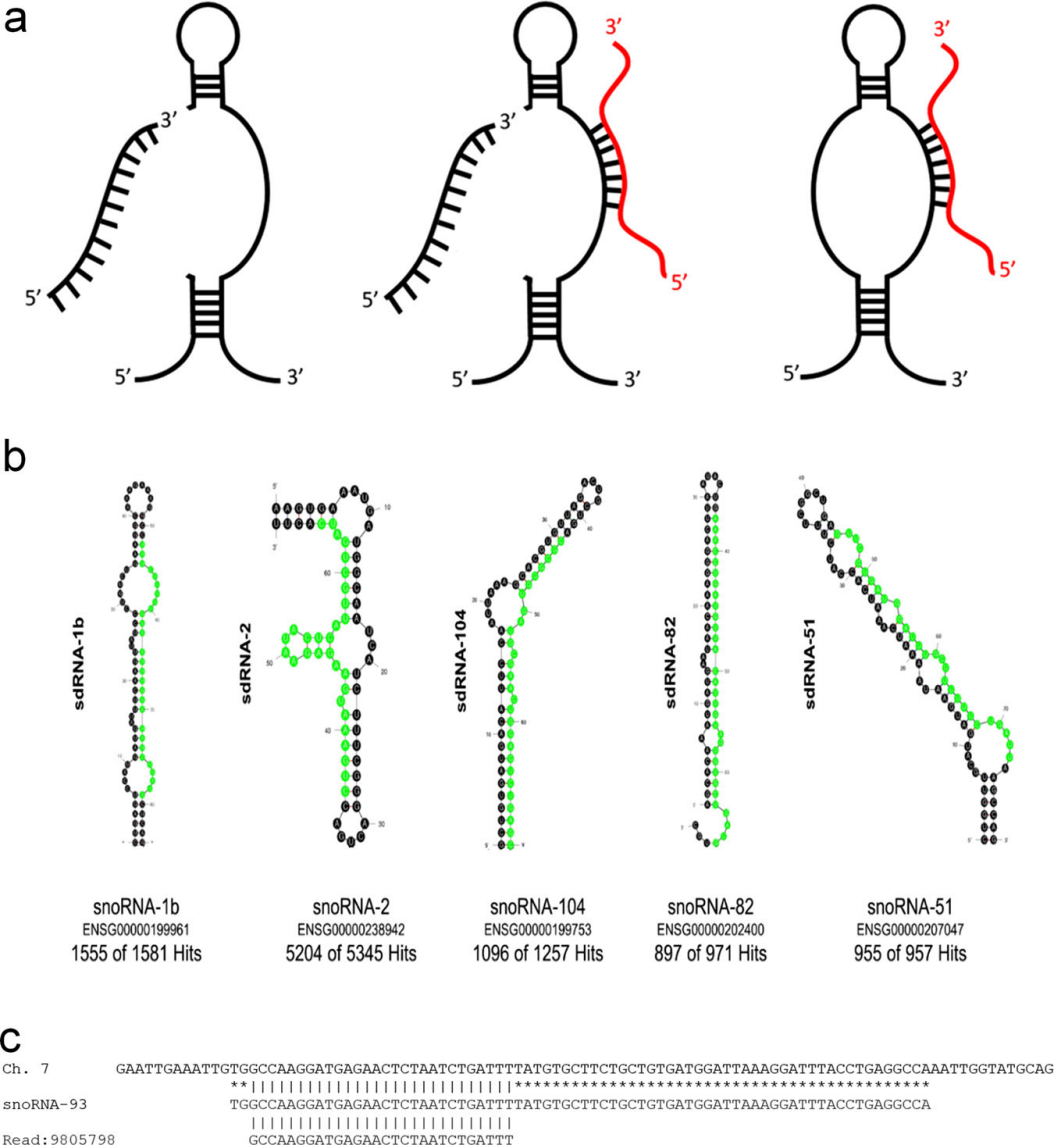
SdRNAs are specifically processed from annotated snoRNA loci. **a** Transcripts arising from various annotated snoRNA loci have now been definitively shown to participate in at least two distinct noncoding RNA regulatory pathways. Individual loci can produce snoRNAs functioning exclusively as either a traditional RNA editor (*right*) or as a functional miRNA precursor (*left*) while some loci have now been confirmed to produce transcripts at times engaging in both types of noncoding RNA regulation (*center*) (reviewed in ref. 5). MiRNA-like excision products are illustrated in *black* (*left* and *center*) as excision products of primary transcript. Complementary RNA editing targets are shown in *red* (*right* and *center*). **b** The most thermodynamically stable secondary structures of putative sdRNA producing snoRNAs with sdRNA sequences highlighted in green as calculated by Mfold.^53^ Common name and Ensembl gene id for putatively processed snoRNAs are listed below corresponding structures. “Hits” refer to the number of times fragments of putative sdRNA producing snoRNAs perfectly aligned to small RNA-seq reads from individual SRA datasets. Numbers preceding total numbers of hits correspond to the number of times positions highlighted in green (putative sdRNAs) perfectly aligned to small RNA-seq reads (e.g., 1555 of 1581 small RNA reads aligning to snoRNA-1b corresponded to the sequence highlighted in *green*). **c** Alignment between the human genome (GRCh38:7:22856601:22856699:1) (*top*), snoRNA-93 (ENSG00000221740) (*middle*), and next generation small RNA sequence read (*bottom*) obtained by Illumina sequencing of MDA-MB-231 RNA is shown. All sequences are in the 5′ to 3′ direction. An *asterisk* indicates base identity between the snoRNA and genome. *Vertical lines* indicate identity across all three sequences

Mature microRNAs (miRNAs) are noncoding RNAs consisting of 18–25 nucleotides that associate with the RNA-induced silencing complex (RISC) and bind to specific mRNA targets in their 3′ untranslated regions (3′ UTRs), ultimately resulting in gene suppression through the translational repression or cleavage of their bound mRNAs. Widely perceived as being distinct from and wholly unrelated to miRNAs, small nucleolar RNAs (snoRNAs) are localized within the nucleolus and have long been characterized as molecular guides for sequence-specific modifications to ribosomal RNAs (rRNAs) and small nuclear RNAs (snRNAs).^1, 2^ Strikingly, despite their well characterized roles in guiding these modifications, nearly one-third of snoRNAs show no complementarity to known modified positions in rRNAs or snRNAs, indicating that these “orphan snoRNAs”^3^ either: (I) guide modifications to an entirely different type of RNA not yet characterized as being snoRNA edited or (II) function in other measures that have yet to be realized.^3^ Intriguingly, there is now extensive evidence that many snoRNAs are processed into short stable miRNA-like fragments called small nucleolar RNA-derived RNAs (sdRNAs) (reviewed in ref. 4, 5). Surprisingly, this phenomenon is not simply limited to orphan snoRNAs, indicating that many snoRNAs may perform more than one distinct function^6, 7^ (Fig. 1a).

The first account of a snoRNA acting as a precursor for a functional sdRNA was reported in 2008. In this report, H/ACA-box snoRNA ACA45 was characterized as producing a sdRNA in a Dicer-dependent manner, and what’s more, the authors definitively confirmed this sdRNA could repress the expression of a gene encoding Cyclin-dependent kinase 11a (CDK11A or CDC2L2).^6^ Soon after, additional studies reported sdRNAs exhibiting miRNA-like properties including Dicer-dependent processing, Argonaute (Ago) protein association (an essential RISC component), and efficient mRNA silencing.^6, 8–13^ Furthermore, it was revealed that the expression of many sdRNAs differed among cell types—a hallmark of miRNA regulatory activity.^11^

Also of note, several reports have recently provided evidence suggesting that snoRNA dysregulations can actively contribute to carcinogenesis. For example, SNORD50 was first proposed to be a candidate tumor-suppressor gene in prostate cancer after researchers found a two-basepair homozygous deletion in the genetic locus of SNORD50 in 30 prostate cancer cell lines. Excitingly, ectopic expression of SNORD50 significantly reduced colony formation in these prostate cancer cells.^14^ A more recent report showed SNORD50 was also involved in the development and progression of breast cancer further supporting the role of SNORD50 in regulating malignant transformation and progression.^15^ Additional snoRNAs with tumor suppressive functions include SNORA42 in non-small-cell lung cancer,^16^ SNORA47^17^ and SNORD76 in glioblastoma,^18^ SNORA47^19^ and SNORD113-1 in hepatocellular carcinoma,^20^ SNORA23 in human pancreatic ductal adenocarcinoma,^21^ and SNORD44 in head and neck squamous cell carcinoma and breast cancer.^22, 23^ Collectively, these reports provide evidence that snoRNAs are functionally important in cancer and that, like miRNAs, snoRNAs can function as oncogenes or tumor suppressors. Moreover, these reports suggest the existence of unrecognized non-canonical snoRNA activities and raises the possibility that the frequent processing of snoRNAs into sdRNAs may prove pathologically relevant.

Interestingly, a recent study comparing the small RNA transcriptome of normal and malignant prostate tissue reported that sdRNAs not only make up at least one-third of all identified differentially expressed small RNAs but also display stronger differential expression than miRNAs. Strikingly, expression of one of the differentially expressed snoRNAs (SNORD78) and its sdRNA was associated with a subset of patients that developed metastatic disease.^24^ Also of note, two separate reports have recently suggested active roles for sdRNAs in breast cancer pathogenesis. The first of these described a group of polycistronic miRNA-like sdRNAs that are transcriptionally repressed by p53 via SNHG1. The authors of this work go on to show that the most highly expressed of these sdRNAs, sno-miR-28, actively represses the p53-stabilizing gene (TAF9B), thereby creating a regulatory feedback loop charged with controlling p53 stability. In addition, the authors also found that SNHG1, SNORD28, and sno-miR-28 were all significantly upregulated in select breast tumors and that sno-miR-28 overexpression enhanced breast epithelial cell proliferation.^25^ More recently, Krishnan et al. performed a comprehensive, large scale next generation small RNA sequencing analysis examining over 100 unique breast malignancies, and identified 64 snoRNAs harboring piwi interacting RNAs and/or miRNAs predicted to target genes involved in tumorigenesis.^26^ Following completion of this study, the authors reported 13 snoRNAs whose expression significantly correlated with overall survival, clearly demonstrating the potential of snoRNAs to serve as prognostic markers for breast cancer.^26^

These findings highlight the need for additional in-depth examinations of snoRNAs and their sdRNAs in these and other cancer subtypes as the recently emerging body of evidence strongly suggests that sdRNAs play a greater role in tumorigenesis then what is currently appreciated. As such, we recently elected to compare the snoRNA and sdRNA expressions of two well-characterized breast cancer cell lines: primary MCF-7 and metastatic MDA-MB-231. Hypothesizing that sdRNAs significantly differentially expressed between these two classic breast cancer models might well represent uncharacterized contributors to their pathological differences, we began our analysis by performing a comprehensive small RNA-seq-based examination of their transcriptomes resulting in the identification of 13 snoRNAs with markedly different abundances (Supplementary Table 1). Excitingly, we found that one of these, snoRNA-93 (aka HBII-336) was precisely processed into a smaller RNA (sdRNA-93) that was markedly overexpressed in MDA-MB-231 cells as compared to MCF-7s. We found that sdRNA-93 suppression decreases breast cancer cellular invasion, whereas increasing its abundance reciprocally enhances invasion. As such, we suggest the significantly higher levels (>75x) of sdRNA-93 in MDA-MB-231s as compared to MCF-7s likely directly contributes to the characteristically higher invasiveness of MDA-MB-231 cells. Importantly, in addition to describing the phenotypic consequences of manipulating cellular sdRNA-93 levels, the current study also describes a regulatory target of sdRNA-93, the sarcosine metabolism-related gene Pipox, whose expression has previously been shown to strongly correlate with specific breast cancer subtypes and prognosis.^27^ In summary, this work successfully identifies a specific sdRNA (sdRNA-93) contributor to breast cancer pathology further emphasizing the relevance of this relatively new form of noncoding RNA regulator to malignancy.

## Results

### Evaluation of the prevalence of sdRNA production

Prior to initiating our comparison of the MDA-MB-231 and MCF-7 small RNA transcriptomes we examined the relationship between snoRNAs and miRNAs in silico. To address the extent to which snoRNAs can function as precursors for functional miRNAs (Fig. 1a), we began by performing a comprehensive analysis of the sequence relatedness between all known snoRNAs and miRNAs. Interestingly, we identified 42 definitively aligning to 56 unique miRNA hairpins (Supplementary Table 2). Strikingly, these snoRNA::miRNA hairpin alignments average 95.4% identity over 68.8 nts and strongly support several conserved evolutionary relationships between snoRNAs and miRNAs.^10^ After confirming snoRNAs and miRNA hairpins display strong sequence similarity, we next examined the prevalence of sdRNAs in publically available small RNA next generation sequencing Sequence Read Archive (SRA) files. After screening 18 deep-sequencing transcriptome profiles involving nine species, we found miRNA-like fragments aligning to 38 of our 42 miRNA-like snoRNAs (Supplementary Table 3). The length of these fragments varied between 21 and 29 nts; although the majority were ≤25 nts in length. Importantly, we found snoRNA fragments mapping to larger snoRNA precursors did not likely represent degradation products as >90% of putative sdRNA sequences were specifically excised from snoRNA precursors at fixed positions (Fig. 1b). In addition, tripartite alignments were generated to confirm sdRNA origins through visualizing sdRNA and corresponding parental snoRNA sequence alignments to genomic loci (Fig. 1c, Supplementary Table 4).

### Overexpressed snoRNAs in metastatic breast cancer are processed into miRNAs

As a growing body of evidence now suggests that sdRNAs play a greater role in tumorigenesis then what is currently appreciated, we next elected to compare the snoRNA and sdRNA expressions of two well-characterized breast cancer cell lines: primary MCF-7 and metastatic MDA-MB-231. Hypothesizing that sdRNAs differentially expressed between these two breast cancer models might represent uncharacterized contributors to their pathological differences, we began by performing comprehensive small RNA-seq of their transcriptomes. In total, 23,219,312 and 21,092,404 high quality reads were generated for MCF-7 and MDA-MB-231, respectively. Representing the two most widely utilized breast cancer cell lines, primary MCF-7 and metastatic MDA-MB-231, have been used in over half of all reported breast cancer studies.^28^ Since these cell lines differ in several well established ways in terms of morphology, invasiveness, and physiological responses (reviewed in ref. 29), we selected these models to perform a RNA-seq analysis specifically examining their snoRNA expression levels and sdRNA pervasiveness in order to identify uncharacterized gene candidates responsible for their phenotypic differences. Given the well documented relationship linking dysregulated miRNAs with tumorigenesis and metastasis,^30–32^ we reasoned that sdRNAs overexpressed in MDA-MB-231 (relative to MCF-7) cells might specify divergent malignant traits between these samples. Excitingly, our comparison of the snoRNA expression profiles of these two breast cancer models did result in the successful identification of 13 snoRNAs expressed at markedly higher levels (≥7.5x) in MDA-MB-231s as compared to MCF-7s (Supplementary Table 1). Furthermore, we find miRNA-like fragments derived from all 13 of these snoRNAs in MDA-MB-231s, and that 10 of the 13 are found in complex with the miRNA-associating protein Ago in available SRA datasets (data not shown) strongly suggesting their active involvement in the RNAi pathway. Furthermore, we also found five of these sdRNAs were preferentially processed (≥5.5x) from their parental snoRNAs in MDA-MB-231s as compared to MCF-7s (Supplementary Table 5).

### Confirming the efficacy of tools designed to manipulate sdRNA-93 levels

**Fig. 2:**
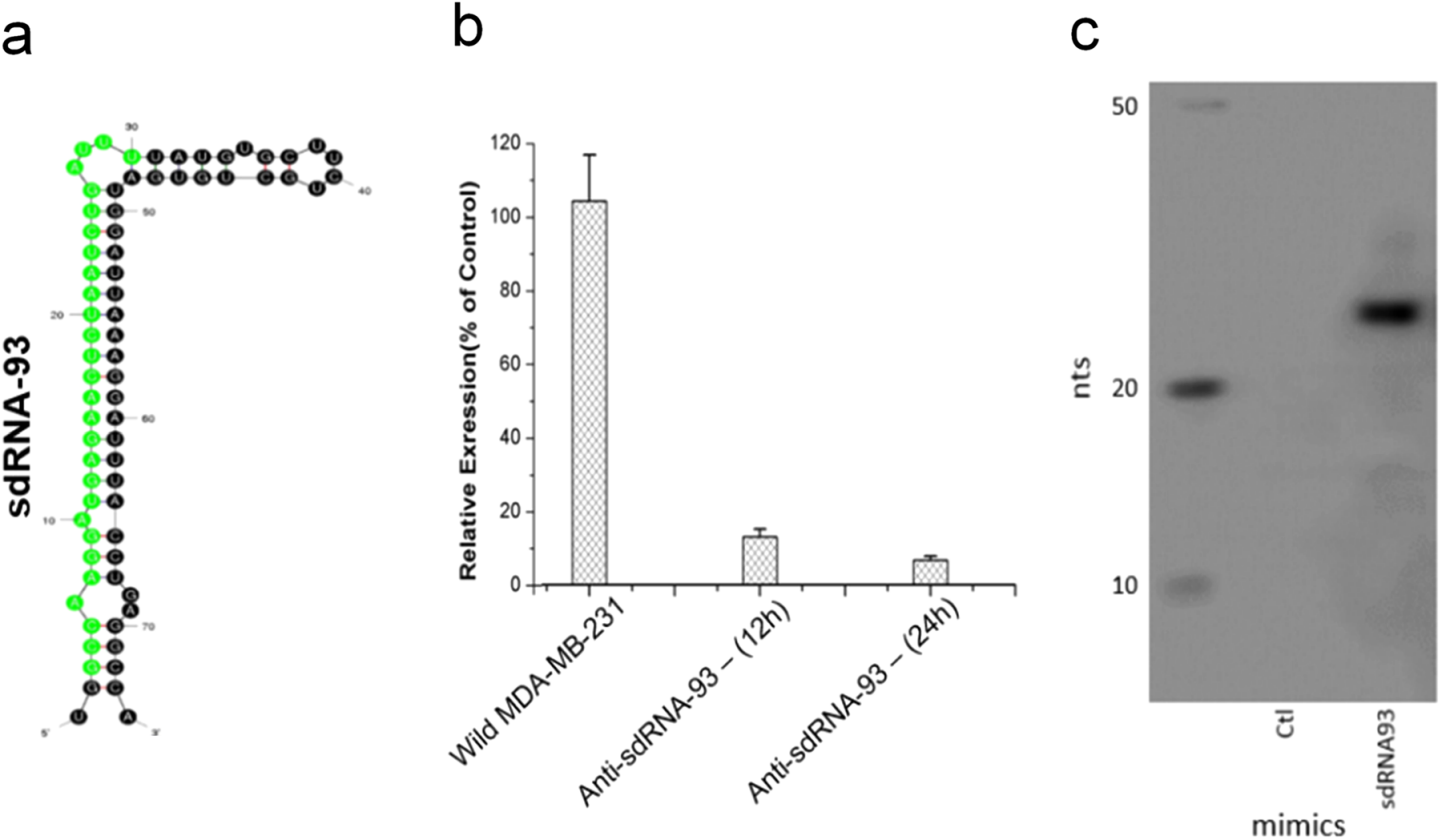
Tools for manipulating sdRNA-93 expression. **a** The most thermodynamically stable secondary structure of snoRNA-93 as calculated by Mfold.^53^ Predominately excised sdRNA-93 sequence is highlighted in *green*. **b** Knockdown of sdRNA-93 by transient transfection of MDA-MB-231 cells with custom inhibitor (antisense to sdRNA-93) sequence depicted in *green* in **a** as determined by RT-qPCR (*n* = 3). **c** Small transcript northern blot confirming the over expression of sdRNA-93 following transfection of MDA-MB-231 cells with custom mimic (identical to sdRNA-93 sequence depicted in *green* in **a** vs. scrambled control (ctl))

Next, in order to directly determine if a specific sdRNA can regulate phenotypical aspects of malignancy, we elected to focus on sdRNA-93 for in depth experiments as: (i) sdRNA-93 was the most highly overexpressed sdRNA (≥75x) in our MDA-MB-231 RNA-seq data as compared to MCF-7s (independently verified by quantitative PCR (qPCR)—data not shown) (Supplementary Table 5), and (ii) a previous study has shown that sdRNA-93 possesses miRNA-like silencing properties via luciferase assays.^6, 12^ To achieve this, we began by designing and commercially synthesizing custom miRNA mimics and inhibitors based on sdRNA-93 (Fig. 2a) thereby allowing us to directly examine the phenotypic consequences of manipulating sdRNA-93 levels in culture. Importantly, qPCR analysis confirmed MDA-MB-231 cells transfected with anti-sdRNA-93 show an ~90% reduction in sdRNA-93 precursor SNORD-93 expression at 12 and 24 h as compared to controls (Fig. 2b), and conversely, cells transfected with our sdRNA-93 mimic show a visibly striking increase in sdRNA-93 expression at 24 h as determined by small transcript northern blotting (Fig. 2c).

### Phenotypic effects of manipulating sdRNA-93 levels in MDA-MB-231s

**Fig. 3:**
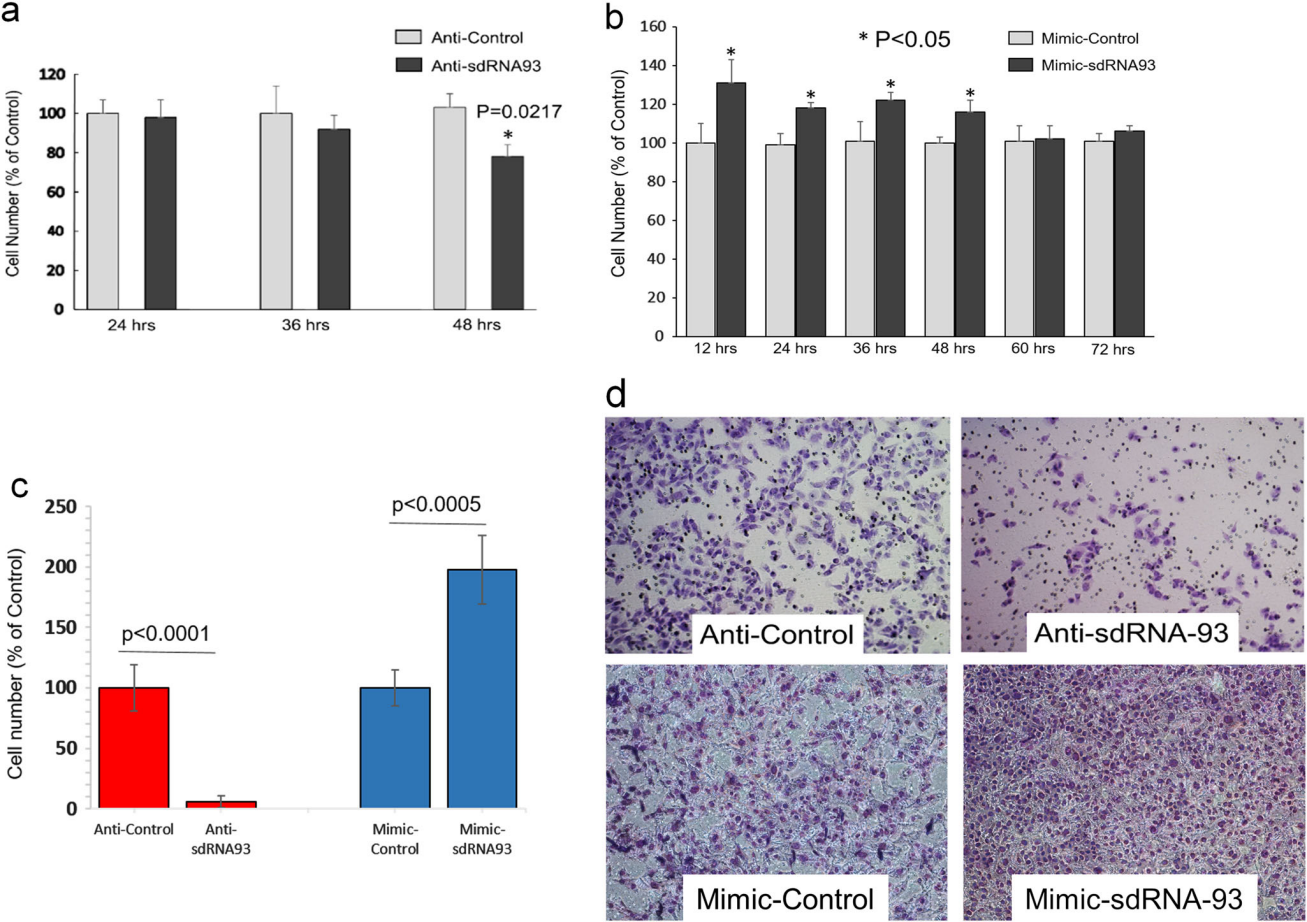
Effects of altered sdRNA-93 expression on MDA-MB-231 cells. **a** MDA-MB-231 cell lines were transfected with anti-sdRNA-93 or a scrambled control RNA. Cell counts performed at 24, 36 and 48 h. **b** MDA-MB-231 cell lines were transfected with mimic-sdRNA-93 or a scrambled control RNA. Cell counts performed at 12, 24, 36, 48, 60 and 72 h. **c** Invasion assays were performed to evaluate the effect of manipulating sdRNA-93 levels on MDA-MB-231 cell invasion. Cell invasion was determined by embedded cell count at 48 h post transfection (*n* ≥ 3). **d** Representative invasion assays quantified in **c**

Since sdRNA-93 had previously been shown to function like a miRNA^6, 12^ and since we found it to be significantly more highly expressed (≥75x) in metastatic MDA-MB-231s as compared to primary MCF-7 cells, we elected to begin our phenotypic examination of the effects of manipulating sdRNA-93 levels in culture in MDA-MB-231s. We began by first examining the effects of silencing sdRNA-93 on MDA-MB-231 cell growth by performing cell counts at 24, 36, and 48 h post anti-sdRNA-93 transfection. Of note, we found cell proliferation was reduced ~20% at 48 h for anti-sdRNA-93 transfected cells as compared to cells transfected with nonspecific control (*p* = 0.0217) (Fig. 3a) suggesting perhaps a somewhat limited role for sdRNA-93 in tumor cell growth. Importantly, however, we also observed the reciprocal effect when we conversely transfected MDA-MB-231 cells with sdRNA-93 mimic finding cellular proliferation increased by 20–30% for the first 48 h post transfection for sdRNA-93 mimic transfected cells as compared to cells transfected with nonspecific control (*p* < 0.05) (Fig. 3b).

Following this, we next examined if manipulating sdRNA-93 levels might influence tumor cell migration or invasion. We began by assessing the effects of altered sdRNA-93 levels on cellular migration by performing standard migration scratch assays. Of note, we found no significant correlation between sdRNA-93 expression and cellular migration (Supplementary Fig. 1). That said, we next evaluated the effects of manipulating sdRNA-93 levels on tumor invasion. To achieve this, MDA-MB-231 cells transfected with anti-sdRNA-93 were plated on matrigel-coated inserts and exposed to a chemoattractant for 48 h then noninvasive cells removed from the surface of the membrane allowing for invading cells to be stained and invasiveness quantified. Excitingly, and in stark contrast to our migration assays, we found sdRNA-93 silencing reduced MDA-MB-231 cellular invasion by >90% at 48 h as compared to cells transfected with scrambled control, and conversely that sdRNA-93 over expression could reciprocally increase MDA-MB-231 cellular invasion by >100% in the same amount of time (Fig. 3c, d). We suggest these results strongly indicate that sdRNA-93 primarily regulates invasion (vs. proliferation or migration), and links a specific sdRNA (sdRNA-93) to an aggressive phenotype characterizing MDA-MB-231 cells.

### Phenotypic effects of manipulating sdRNA-93 levels in MCF-7s

**Fig. 4:**
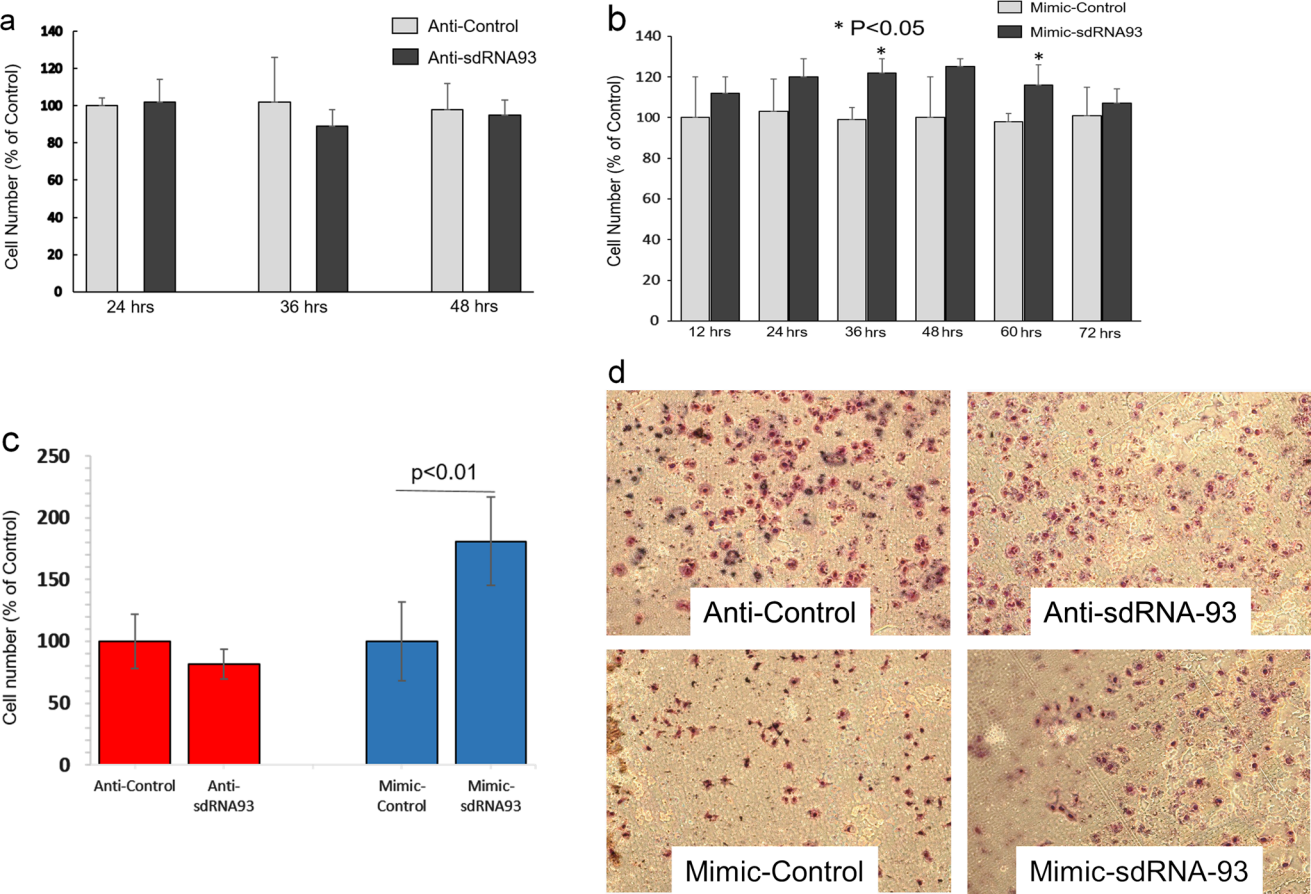
Effects of altered sdRNA-93 expression on MCF-7 cells. **a** MCF-7 cell lines were transfected with anti-sdRNA-93 or a scrambled control RNA. Cell counts performed at 24, 36 and 48 h. **b** MCF-7 cells were transfected with mimic-sdRNA-93 or a scrambled control RNA. Cell counts performed at 12, 24, 36, 48, 60 and 72 h. **c** Invasion assays were performed to evaluate the effect of manipulating sdRNA-93 levels on MCF-7 cell invasion. Cell invasion was determined by embedded cell count at 48 h post transfection (*n* ≥ 3). **d** Representative invasion assays quantified in **c**

We next similarly examined of the effects of manipulating sdRNA-93 levels in culture in MCF-7. We began by first examining the effects of silencing sdRNA-93 on MCF-7 cell growth by performing cell counts at 24, 36, and 48 h post anti-sdRNA-93 transfection. Of note, unlike what we found in MDA-MB-231s, we found cell proliferation was not significantly reduced during the first 48 h following anti-sdRNA-93 transfection of MCF-7s (Fig. 4a). However, when we conversely transfected MCF-7 cells with sdRNA-93 mimic we found cellular proliferation somewhat increased at 36 and 60 h post transfection for sdRNA-93 mimic transfected cells as compared to cells transfected with nonspecific control (*p* < 0.05) (Fig. 4b). Similarly, when we next evaluated the effects of manipulating sdRNA-93 levels on tumor invasion, we found comparable data variance to what was observed in MDA-MB-231 cells and that while sdRNA-93 silencing had little to no effect on cellular invasion, sdRNA-93 over expression instead led to a striking increase (~80%) in MCF-7 cellular invasion (Fig. 4c, d). Importantly, these results strongly suggest endogenous sdRNA-93 expression does not participate in the regulation of MCF-7 cellular invasiveness.

### Pipox is regulated by sdRNA-93

**Fig. 5:**
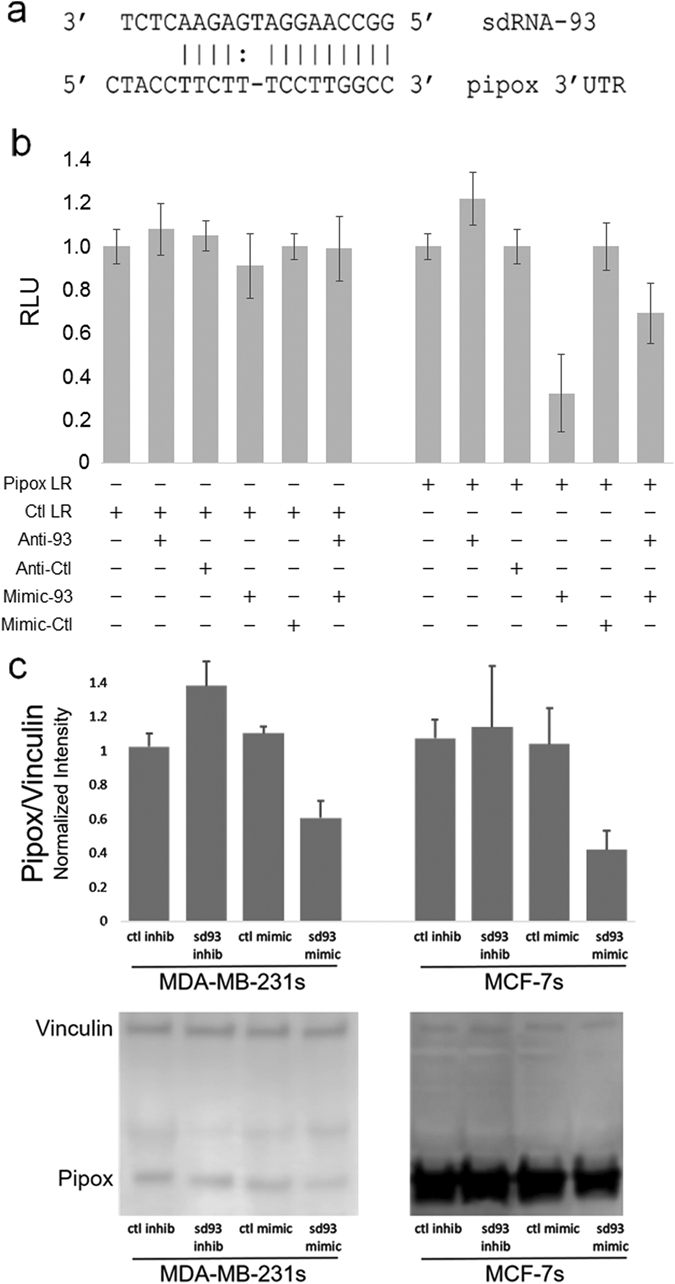
Pipox is an endogenous sdRNA-93 target. **a** Alignment of the putative target site in the Pipox 3′UTR with sdRNA-93. **b** Pipox LR is specifically repressed by sdRNA-93 in HEK293 transient transfections. Luciferase assays (*n* = 4) of HEK293 lysates after cotransfection of Pipox LR, Ctl LR, Anti-93, Anti-Ctl, Mimic-93 and / or Mimic-Ctl as indicated (+ or −). Transfections were normalized to respective LR alone. Pipox LR, luciferase reporter containing sdRNA-93 target site from Pipox 3′UTR; Ctl LR, luciferase reporter containing scrambled sdRNA-93 3′UTR target sites; Anti-93, sdRNA-93 inhibitor; Anti-Ctl, control inhibitor; Mimic-93, sdRNA-93 mimic; Mimic-Ctl, control mimic; RLU, relative light units. **c** Representative western blots of MDA-MB-231 (*left*) and MCF-7 (*right*) breast cancer cells transfected with sdRNA-93 inhibitor, sdRNA mimic or scrambled control. Pipox and Vinculin (control) blots are shown. Graphs indicate the relative ratio of Pipox to Vinculin as normalized to nontransfected control. (*n* ≥ 3)

Since individual miRNAs (and likely sdRNAs) target multiple mRNAs, and since small RNAs are typically only partially complementary to their mRNA target sequences, it has proven exceptionally difficult to identify legitimate endogenous miRNA targets. That said, a number of algorithms have been developed that can be utilized to predict and identify potential miRNA target sequences (e.g., MiRanda, TargetScan, PicTar, and OrbId^33–39^). While these distinct algorithms employ a wide-ranging variety of strategies for target prediction, such as target site conservation, seed complementarity, and thermodynamic stability, each algorithm carries its own unique advantages and limitations and many routinely predict hundreds of putative targets for individual miRNAs. As such, in an attempt to simplify the prediction of endogenous targets of sdRNA-93, we elected to limit putative targets to target genes (1) predicted by multiple algorithms and (2) found to be expressed in our RNA-seq analyses. Of note, we found employing this streamlined methodology for identifying likely endogenous targets of sdRNA-93 readily yielded a marked candidate for sdRNA-93 regulation, Pipox (Fig. 5a), and that sdRNA-93 mimic transfection of HEK-293 cells could silence expression from a standard *Renilla* luciferase reporter ^40^ containing this putative Pipox target site by more than 60% as compared to controls (Fig. 5b). Interestingly, Pipox is a sarcosine metabolism-related protein whose expression levels have recently been reported to significantly correlate with distinct molecular subtypes of breast cancer.^27, 41–43^ Excitingly, we found MDA-MB-231 breast cancer cells treated with sdRNA-93 inhibitor strongly suggest that Pipox represents a legitimate endogenous target of sdRNA-93 as our initial western blotting demonstrated a dose responsive increase in Pipox expression accompanying sdRNA-93 inhibition (Supplementary Fig. 2). That said, subsequent western blotting analyses not only further confirmed that decreasing sdRNA-93 levels results in increased Pipox expression, but also demonstrated that increasing sdRNA-93 cellular levels conversely results in decreased Pipox expression in MDA-MB-231s. That said, while we found that increasing sdRNA-93 levels similarly resulted in decreased Pipox expression in MCF-7s, in contrast to MDA-MB-231s, we found decreasing sdRNA-93 levels in MCF-7s had little to no effect on Pipox expression (Fig. 5c) and speculate this is likely the result of a combination of low endogenous levels of sdRNA-93 (Supplementary Tables 1, 5) and a strikingly high rate of Pipox expression (Fig. 5c) in MCF-7 cells.

Collectively, when taken with our culture analyses, these experiments strongly suggest that regulating the expression of Pipox represents a legitimate endogenous function of sdRNA-93 in vivo (Fig. 5).

### SdRNA-93 expression correlates with Luminal B Her2+tumors

**Fig. 6:**
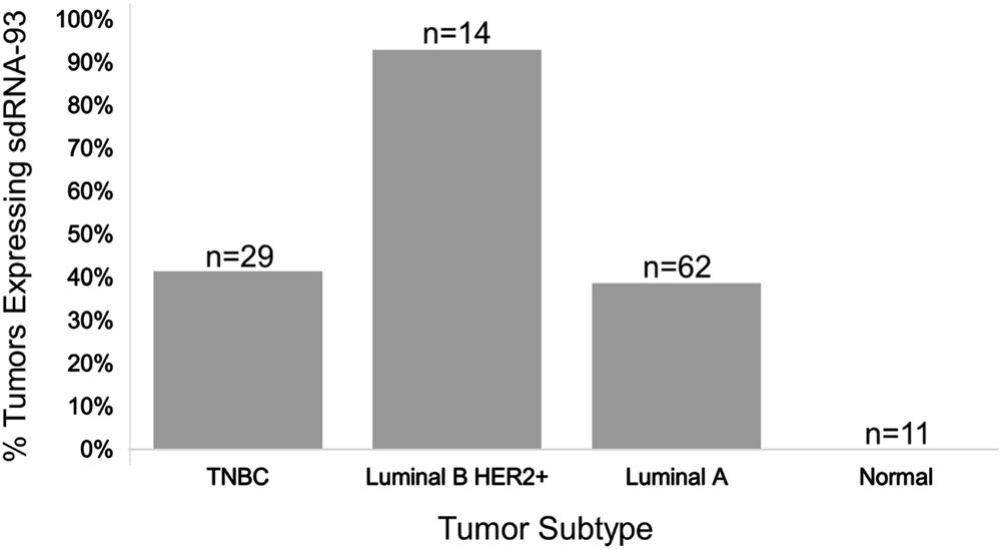
SdRNA-93 expression in distinct breast cancer subtypes. In all, we determined sdRNA-93 expressions in 116 small RNA-seq files corresponding to 116 breast cancer patient tumors and/or normal breast tissue controls. SnoRNAs were counted as significantly expressed if counts were ≥250 counts/million reads. The number of sequencing files/patient samples corresponding to a particular subtype are indicated (*n* = ). Percentage was calculated as the number of samples significantly expressing sdRNA-93/total number of samples of that subtype

In a recent report, Krishnan et al.^26^ performed an extensive computational analysis of over 100 publicly available small RNA-seq SRA files each corresponding to a unique breast cancer patient tumor classified as either triple negative breast cancer (TNBC), Luminal A, or Luminal B Her 2+subtype or normal tissue control leading to the successful characterization of several full length snoRNAs with likely prognostic value for breast cancer. In order to similarly examine the expression of sdRNA-93 in various breast cancer subtypes, we elected to obtain the same SRA data sets utilized by Krishnan et al.^26^ along with several additional controls. Excitingly, as depicted in Fig. 6, we do find marked differences in the expression of sdRNA-93 between breast cancer subtypes, and a definitive correlation between sdRNA-93 expression and the Luminal B HER2+subtype. In all we found 12 of 29 (41.4%) TNBC tumors, 24 of 62 (38.7%) Luminal A tumors, 13 of 14 (92.9%) Luminal B HER2+patient tumors and 0 of 11 normal tissue controls measurably expressed sdRNA-93.

## Discussion

Breast cancer is the most common female malignancy in most European and North American countries and the leading cause of female cancer mortality. Strikingly, defined by distinct characteristics in terms of morphology, invasiveness and physiological responses, primary MCF-7 and metastatic MDA-MB-231 breast cancer cells are involved in over half of all primary breast cancer reports.^28^ Although both MCF-7 and MDA-MB-231 are adenocarcinomas (cancers of the breast epithelium that originated in the mammary gland) they are characterized by distinct differences in cellular morphology, activity and gene expressions. The MCF-7 breast cancer line was derived from an in situ carcinoma, where the cancerous cells had not yet invaded surrounding tissues. These cells are weakly invasive, and luminal epithelial-like. They are also hormone responsive as they express estrogen and progesterone receptors (PRs) and the presence of estrogen stimulates their non-specific proliferation. Conversely, the highly invasive, fibroblast-like MDA-MB-231 line was derived from a metastatic carcinoma and is not hormone sensitive as it is a TNBC, lacking estrogen receptor, PR and human epidermal growth factor receptor 2 (HER2) expression. Relative to MCF-7 cells, MDA-MB-231 cells grow faster and are more resistant to drug therapies. Clinically, the cancer of the MDA-MB-231 cell line is harder to treat, and noticeably less aggressive methods are necessary when treating MCF-7 cells.^44^

Importantly, we find sdRNA-93 is expressed in MDA-MB-231s at >75x its expression in MCF-7s. Additionally, increasing or decreasing its expression in MDA-MB-231s reciprocally alters cellular proliferation and invasion (Fig. 3), and while increasing sdRNA-93 expression similarly enhances MCF-7 invasion, transfecting MCF-7s with anti-sdRNA-93 has little to no effect (Fig. 4). Collectively, these results indicate that sdRNA-93 directly contributes to MDA-MB-231 tumorigenicity, and that elements of their characteristic phenotypic differences can be directly attributed to their strikingly contrasting levels of sdRNA-93 expression (Supplementary Table 5).

Of note, in addition to sdRNA-93 mimic transfections corroborating the results of our anti-sdRNA-93 analyses through directing reciprocal phenotypic consequences (Figs. 3–5), these results also confirm that sdRNA-93 is the specific RNA molecule whose manipulation directly conveys the observed effects on cellular proliferation and invasion as opposed to being a consequence of altering endogenous levels of the full length parental snoRNA-93. While the effects of anti-sdRNA-93 could conceivably be due to an inhibition of full length snoRNA-93, the effects of the mimic can only be contributed to “excised” sdRNA-93 (Fig. 2a). These results therefore demonstrate that our observed phenotypic consequences are due to manipulating levels of the miRNA-like sdRNA-93, and suggest a functional role for this sdRNA in breast pathology. That said, further studies will be required to fully establish the phenotypes associated with the expression of this sdRNA in normal and malignant breast tissues as well as to characterize additional RNAi regulatory targets of this sdRNA.

Of note, our initial target prediction analyses readily identified Pipox as a likely regulatory target of sdRNA-93, and our subsequent experimental analyses confirm that sdRNA-93 can directly regulate the Pipox 3′UTR and that endogenous Pipox expression directly correlates with that of sdRNA-93 (Fig. 5). That said, although our initial target predictions originally identified Pipox as the only highly scoring putative target of sdRNA-93, we recently reconfigured our target prediction strategy resulting in the identification of two other putative sdRNA-93 targets (AbI Interactor 2 (Abi2) and Ring Finger Protein 4 (RNF4)). We find both of these biologically interesting, robustly expressed in our MDA-MB-231 RNA-seq data, and that luciferase reporters containing their putative target sequences are similarly repressed by cotransfection with sdRNA-93 mimic (Supplementary Figs. 3, 4). In addition to this, in light of the established role of Pipox in sarcosine metabolism,^43^ we also recently elected to examine if sdRNA-93 could regulate the expressions of the other principle genes involved with sarcosine metabolism. To address this, we performed qPCRs assessing the effects of manipulating sdRNA-93 levels on the expressions of two other principle sarcosine metabolism related genes: glycine N-methyltransferase (GNMT) and sarcosine dehydrogenase (SARDH).^43^ In contrast to the ability of sdRNA-93 to regulate Abi2 and RNF4 target sequence reporters, we find sdRNA-93 mimic transfection does not significantly effect GNMT or SARDH expression in MDA-MB-231s or MCF-7s (data not shown). Although more detailed experimentation will clearly be required to fully characterize sdRNA-93’s endogenous regulations, these data suggest that sdRNA-93 may well regulate more than Pipox.

Also of note, due to the inherent limitations of qPCRs for quantifying mature miRNAs and miRNA-like molecules (particularly those that have been chemically modified like the inhibitors utilized in this study),^45^ for this study we elected to perform small transcript northern blotting to evaluate sdRNA-93 expression following mimic transfection. Importantly, MCF-7s and MDA-MB-231s were identically transfected with the same concentration of sdRNA-93 mimic, and as illustrated in Fig. 2c, mimic transfection results in robust cellular levels of sdRNA-93 RNA many fold above the endogenous levels of sdRNA-93 in either MDA-MB-231s or MCF-7s. As such, we suggest mimic transfections essentially constituted a cellular saturation of available sdRNA-93 in both cell lines far above normal physiological expression. That said, we observed no evidence suggesting the high level of cellular expression corresponding to mimic transfection results in any notable cellular toxicity to either breast cancer cell line (see Figs. 3b, 4b) yet comparably enhances the invasion of both (Figs. 3c, 3d, 4c, 4d), suggesting that sdRNA-93 targets are saturated at the levels found in MDA-MB-231s and that there is little to no off target effect toxicity associated with sdRNA-93 overexpression. Furthermore, as a complement to our small transcript northern blotting and similarly in lieu of performing qPCRs on mature sdRNAs, we additionally elected to perform qPCRs quantifying cellular levels of full-length snoRNA-93 following inhibitor transfection. Notably, these analyses confirmed efficient knockdown of full-length snoRNA-93 (~80%) (Fig. 2b), and we suggest this likely correlates with a concurrent decrease in available sdRNA-93, especially in light of the reciprocal phenotypic results we observe following mimic transfection (Figs. 3, 4).

Finally of note, despite characterizing several snoRNAs with likely prognostic value to breast cancer using the same data sets we utilized to identify the correlation between sdRNA-93 and the Luminal B Her2+breast tumor subtype depicted in Fig. 6, the initial analyses performed by Krishnan et al.^26^ did not identify any significant correlation between snoRNA-93 expression and breast malignancy. While our analyses both observe robust expression of SNORD93, Krishnan et al. did not report any significant production of sdRNA-93 in these samples. In contrast, our analyses did find sdRNA-93 to be specifically excised from the 5′ end of SNORD93 (or HBII-336) in agreement with and as originally characterized by Brameier et al.^12^. Furthermore, while we find little significant difference in the expression of SNORD93 in these data sets, we do find marked differences in the expression of sdRNA-93 between breast cancer subtypes (Fig. 6) and suggest this indicates that sdRNA expressions can differ markedly from that of their precursors and that their expressions can constitute relevant features in their own right (Supplementary Tables 1, 5).

In conclusion, although human miRNAs were only described in 2001, miRNAs have already become widely recognized as important regulators of cell growth, differentiation, and apoptosis,^30, 46, 47^ and what’s more, several miRNA misexpressions have now been directly identified as the causal events responsible for the initial tumorigeneses leading to an array of oncologies.^48–50^ Accordingly, our preliminary analyses (Supplementary Tables 1–5) and the results directly presented in this report strongly suggest that sdRNAs may similarly represent another commonly overlooked, yet strikingly prevalent, form of noncoding RNA. Importantly, although experimental analyses have previously demonstrated that human snoRNAs undergo cytoplasmic processing and can perform efficient mRNA silencing of *Renilla* reporter constructs,^12^ the examination of sdRNA-93 outlined here represents, to our knowledge, the first ever functional characterization of an endogenous human sdRNA miRNA-like regulation directly contributing to malignancy. As such, in addition to our findings strongly supporting the existence and functional relevance of non-canonical snoRNA activities, they also suggest that sdRNAs may routinely serve critical, currently undescribed, roles in human cancer. In light of this, we now suggest that sdRNAs represent an almost wholly overlooked species of noncoding RNA functionally indistinguishable from traditional miRNAs, and that like miRNAs, sdRNAs may well prove invaluable in deciphering the molecular mechanisms contributing to malignancy and represent entirely new tools for diagnostic and prognostic determination as well as a potentially powerful new class of targets for therapeutic intervention.

## Methods

### MCF-7 and MDA-MB-231 RNA sequencing

Samples of two breast cancer cell lines (MCF-7 and MDA-MB-231) recently authenticated and tested for mycoplasma contamination were collected at 70% confluency and suspended in TRIzol (Invitrogen, Carlsbad, CA) were obtained from colleagues at the Mitchell Cancer Institute and sent to a next-generation sequencing service provider (Otogenetics, Norcross, GA). Sequencing was accomplished using a high throughput Illumina HiSeq2000 machine with a paired-end protocol that generated 100 bp reads. Two sequencing runs for each sample were requested: total RNA-Seq and small RNA-Seq.

### Screen MCF-7 and MDA-MB-231 total RNA-seq data for snoRNAs and sdRNAs

Total RNA sequencing was performed for MCF-7 and MDA-MB-231 to provide reads for all RNA transcripts. In total, 48,336,324 and 76,086,874 raw reads were generated for MCF-7 and MBA-MD-231, respectively. Trimmomatic v0.22 (available at www.usadellab.org) was utilized to remove 5′ and 3′ adapter and Illumina primer sequences from raw reads. Alignments between snoRNAs and Illumina reads were obtained via BLAST+ (available at https://blast.ncbi.nlm.nih.gov/Blast.cgi) with all accepted alignments strictly limited to ≥85% identity over ≥40 bps. Small RNA-Seq was performed for MCF-7 and MDA-MB-231 to provide small RNAs ranging from 17 to 35 nucleotides in length. In total, 23,219,312 and 21,092,404 raw reads were generated for MCF-7 and MDA-MB-231, respectively. Trimmomatic v0.22 was employed to remove 5′ and 3′ adapters and Illumina primer sequences from raw reads. Alignments between snoRNAs and next-generation sequencing data were obtained via BLAST+, and all accepted alignments between sdRNAs and snoRNAs were rigidly defined as ≥16 nts, ≤28 nts, and perfect matches (100% identity). Frequency was calculated (count/million reads) for identified snoRNAs and sdRNAs, and then used to compare expression between MCF-7 and MDA-MB-231. SnoRNAs were only counted as overexpressed if read counts were ≥250 counts/million reads for at least one of the cell lines. Significant overexpression of snoRNAs was defined as being ≥5x overexpressed.

### Search for miRNAs derived from miRNA-like snoRNAs in SRA files

Publicly available next-generation small RNA deep-sequencing libraries were obtained from the NCBI SRA (www.ncbi.nlm.nih.gov/sra/). These include (identifiers are listed in parentheses): *Homo sapiens* bladder epithelia, MCF-7 cells under hypoxia, and HEK293 cells (DRR013038, SRR873389, and SRR651728, respectively); *Mus musculus* hypothalamus (SRR747876); *Pan troglodytes* kidney, liver, brain, and testis (ERR038448, ERR038439, ERR038434, and ERR038443, respectively); *Oryza sativa* seedling argaunaute-4 immunoprecipitation and total RNA-seq (SRR037234 and SRR037238); *Gallus gallus* lymphocytes and Marek’s diseased lymphoma from liver (SRR332255 and SRR332253); *Canis lupus* testis (SRR871530); *Bos taurus* CD14+monocytes (SRR1020382); *Gasterosteus aculeatus* brain (DRR003967); and *Medicago truncatula* root (SRR804914). All accepted BLAST+ alignments between sdRNAs and miRNA-like snoRNAs were restricted to perfect matches (100% identity) aligning to ≥16 nts.

### Quantifying sdRNA-93 expression in small RNA-Seq SRA files

A FASTA file containing the sequence for snoRNA-93 was downloaded from Ensembl Biomart^51^ then transferred to the Alabama Supercomputer Authority (https://www.asc.edu). Similarly, 118 publicly available miRNA-sequencing data sets were obtained from the NCBI Sequence Read Archive (SRA) (www.ncbi.nlm.nih.gov/sra/). These include 114 files of sequenced human breast tissue from patient samples (SRR1982469 to SRR1982582) and 4 files sequenced from HER2+ breast cancers (ERR372263 to ERR372266). As there were only two RNA-seq data sets corresponding to the Luminal B HER2- tumor subtype (SRR1982514 and SRR1982575), they were discarded. Small RNA-seq files were converted into FASTA files from FASTQ files using FASTX (available at hannonlab.cshl.edu/fastx_toolkit) on the Alabama Supercomputer. FASTA files derived from SRA datasets were next blasted against snoRNA-93 with alignments between snoRNA-93 and the SRA files obtained via Basic Local Alignment Search Tool (BLAST+) [2]. All accepted alignments between SRAs and snoRNA-93 were limited to perfect matches (100% identity) aligning to ≥16 nts.^52^ Expression of snoRNA-93 was quantified by dividing the number of accepted alignments by the total number of reads in a given SRA file multiplied by one million.

### RNA isolation and quantitative real-time PCR

#### SNORD93 qPCR

Total RNA was extracted using Trizol reagent (Invitrogen, Carlsbad, CA). Cells were harvested and dissolved in 1 mL of Trizol, and then 200 μL of 1-bromo-3-chloropropane solution was added (Molecular Research Center Inc., Cincinnati, OH) and vortexed. Samples were then subjected to centrifugation at 13,000 rpm at 4 °C for 15 min. Once centrifugation was complete, the supernatant was transferred to a new tube, and an equal volume of isopropanol (Sigma-Aldrich, St. Louis, MO) was added for precipitation. Precipitate pellets were then washed using 75% ethanol, air dried, and dissolved in nuclease-free water for spectrophotometric nucleic acid quantification using a Nanodrop (Thermo Scientific, Worcester, MA). RT-qPCR was performed to confirm the levels of snoRNA-93. Total RNA was utilized for cDNA synthesis using a high capacity cDNA reverse transcriptase kit (Applied Biosystems, Foster City, CA). RT primer (5′-TGGCCTCAGGTAAATCCTTTAATC-3′) and RT-qPCR primers (Forward: 5′-ATCCTGGCCAAGGATGAGAACT-3′and Reverse: 5′-ATCCTGGCCTCAGGTAAATCCT-3′) were ordered from Life Technologies (Carlsbad, CA). RT was performed at 37 °C for 2 h by incubating a 20 μL mixture of 2 μg of total RNA, 2 μM RT primer, 2 μL 10× reverse transcription buffer, 1 μL 100 mM dNTP, 1 μL multiScribe reverse transcriptase and nuclease-free water. RT-qPCR reactions were performed using FastStart Universal SYBR Green Master Rox (Roche, Indianapolis, IN). RT-qPCR reaction, consisting of 10 μL 2× SYBR master mix, 2 μL synthesized forward primer and reverse primer mixture, 1 μL cDNA, and 7 μL nuclease-free water, were incubated for 40 cycles on 7500 real-time PCR System (Applied Biosystems, Foster City, CA). The comparative Ct method was used to compute relative levels of snoRNA-93 by subtracting Ct values of the endogenous control, in our case U6, and comparing to a designed calibrator in a batch of samples. *GNMT and SARDH qPCR*: Total RNA was isolated using TRIzol Plus RNA Purification Kit (Ambion-Invitrogen, Carlsbad, CA), following a developer-recommended protocol. The samples were subsequently treated with DNAse (TURBO DNA-free Kit, Ambion-Invitrogen, Carlsbad, CA) to remove any trace DNA contamination that could interfere with qRT-PCR analysis. RT-qPCR primers: GNMT (Forward: 5′-GGTGGAAGAGGGCTTCAGTGTG-3′ and Reverse: 5′-GTCGAAGGCGGGCT CGTG-3′) and SARDH (Forward: 5′-GGAGCTGGAGGAGGAGACG-3′ and Reverse: 5′-GGTTGGACGCGATGAAGAGG-3′) were ordered from Eurofins Genetics (Louisville, KY). RT-qPCR analysis was performed using USB VeriQuest SYBR Green One-Step qRT-PCR Master Mix with Fluorescein 2× (Affymetrix Inc, USA). The reaction, consisting of 10 μL VeriQuest SYBR Green master mix, 0.2 μL VeriQuest 100× RT Enzyme Mix, 1 μL of forward primer (10 μM) and 1.25 μL of reverse primer (10 μM), 1 μL RNA template, and 7.5 μL nuclease-free water, were ran for 40 cycles on iQ5 Multicolor RT-PCR Detection System (Bio-Rad, Hercules, CA).

### Examination of the phenotypic effects of manipulating snoRNA-93 expression

Antisense oligonucleotide was designed to target snoRNA-93 sdRNA (5′-AAATCAGATTAGAGTTCTCATCCTTGGCT-3′) and ordered as custom IDT^®^ miRNA Inhibitors from IDT (Integrated DNA Technologies, Coralville, IA). Additionally, a scrambled nonspecific oligonucleotide was ordered for a negative control (5′-TCGTTAATCGGCTATAATACGC-3′). Similarly, sdRNA-93 mimic (5′-GCCAAGGATGAGAACTCTAATCTGATTT-3′) and scrambled controls were ordered as custom miRIDIAN mimics from Dharmacon (GE Healthcare Dharmacon, Inc, Chicago, IL). Cell migration, proliferation, and invasion assays were then performed to observe the effects of siRNA-mediated knockdown of snoRNA-93. Human MCF-7 and MDA-MB-231 cell lines were cultured at 37 °C in 25 cm^2^ vented flasks (Corning, Manassas, VA) with DMEM (Corning) supplemented with 10% fetal bovine serum (Corning) and 1% PenStrep (Corning) in a humidified atmosphere at 5% CO_2_. For transient transfections both lines were cultured in 12-well plates and grown to 60% confluency before transfection with mimics or inhibitors using Lipofectamine RNAiMAX (Invitrogen). *Examining cell proliferation*: MDA-MB-231 cells were first transfected with either 100 nmol/l of inhibitor-1, inhibitor-2, or negative control using Lipofectamine (Life Technologies, Carlsbad, CA) according the manufacturers protocol. Cell number was determined by trypan blue staining and manual counting at 24, 36, and 48 h post-transfection. Proliferation was determined as the relative cell number compared with vehicle treated (0.1% DMSO) controls (*n* ≥ 3). *Examining cell invasion*: MDA-MB-231 and MCF-7 transfected cells were used for assessment of migration and invasion using a matrigel invasion chamber kit (BD Bioscience, Sparks, MD). The matrigel coated plates were rehydrated in warm DMEM serum-free medium for 2 h at 37 °C. After removing the medium, cells were suspended in 500 μL blank medium, and then 750 μL chemoattractant (medium with 10% fetal bovine serum) was added to the well chamber. Cells were then incubated for 36 h in 5% CO_2_ at 37 °C. For measurement of invading cells, non-invading cells were removed from the upper surface of the membrane by scraping using cotton swabs and invading cells through the matrigel to the bottom of the insert were fixed with paraformaldehyde and then stained with crystal violet for counting (*n* ≥ 3). Cells were observed and photographed using Nikon Eclipse TE 2000-U (Nikon Instruments Inc., Melville, NY). Ten random fields of view for each well were quantified by counting the cells in each field and averaging the results. *Examining cell migration* : Scratch assays were used to assess migration. Both cell lines were transfected with inhibitors in standard petri dishes (Corning), as described for examining cell proliferation, then grown to 100% confluence. A 1 cm-wide zone was scratched across the center of each dish then pictures taken every 12 h to assess the rate of migration.

### Vector construction

Unless otherwise indicated, PCR amplifications were performed in 40 μl reactions at standard concentrations (1.5 mM MgCl2, 0.2 mM dNTP, 1× Biolase PCR buffer, 0.5 U Taq (Bioline USA, Inc., Randolph, MA), 0.5 uM each primer) and using standard cycling parameters (94 °C—3 min, (94 °C—30 s, 55 °C—30 s, 72 °C—60 s) × 30 cycles, 72 °C—3 min) then cloned into Topo PCR 2.1 (Invitrogen) and sequenced. Control reporter, Ctl LR, was constructed by oligonucleotide primer extension (25 cycles with 10 s extensions) with primers (scramLR) containing 5′ Xho-I and 3′ Not-I restriction enzyme sites immediately flanking sequences corresponding to scrambled Pipox, ABI-2 and RNF4 sdRNA-93 target sites. Antisense reporters, Pipox LR, ABI-2 LR and RNF4 LR, were constructed by standard PCR with primers containing 5′ Xho-I and 3′ Not-I restriction enzyme sites. Following digestion, amplicons were ligated into the Renilla luciferase 3′UTR of psiCheck2 (Promega) vector linearized with Xho-I and Not-I. Reporter assays were performed as previously described^40^ and the presence of an independently transcribed firefly luciferase in these reporters allowed normalization for transfection efficiency. Primer sequences were as follows:

Pipox_LRf, AACTCGAGAAGATGTCTCAGATGAAGGGAGTG;

Pipox_LRr, AAGCGGCCGCAGGCAGAAGAAAAAGGGAGCG;

ABI2_LRf, AACTCGAGAGTACAATGCTGAGCTGTCTGGATTG;

ABI2_LRr, AAGCGGCCGCAGCCACAGATTCTGCAGGAGGAG;

RNF4_LRf, AACTCGAGACCCAGGTCACTGTTGCCCCATGTTC;

RNF4_LRr, AAGCGGCCGCACCCGAAGCATCCAAGCCC;

scramLRf, AACTCGAGTTTTTCCCCCACACAAATCTCTGAACGTCGTGCCATTACTACTCTATCGTAAGCACATC;

scramLRr, AAGCGGCCGCCTATGAACCAGGTTACATGGCGAAGGGAAGGAC TAGATGTGCTTACGATAGAGTAGTAATG.

### Luciferase assays

Human embryonic kidney (HEK293) cell line was obtained from GenLantis (San Diego, CA) and cultured in MEM (Mediatech, Herndon, VA) supplemented with 10% fetal bovine serum (Hyclone, Logan, UT), 25 mg/mL streptomycin and 25 I.U. penicillin (Mediatech). Cells were cultured in a humidified atmosphere with 5% CO_2_ at 37 °C. For luciferase assays, HEK293s were cultured in MEM (10% FBS and 1% PS) in 12-well plates. At 90% confluency, cells were transfected following the Lipofectamine 2000 (Invitrogen, Carlsbad, CA) protocol. At 36 h post transfection, cells were scraped from well bottoms and transferred to 1.5 mL Eppendorf tubes. Eppendorfs were centrifuged at 2000 RCF for 3 min, followed by supernatant aspiration and cell resuspension in 300 μl of PBS. Cells were lysed by freeze thaws and debris removed by centrifuging at 3000 RCF for 3 min. 50 μl of supernatant was transferred to a 96-well MicroLite plate (MTX Lab Systems, Vienna, VA) then firefly and Renilla luciferase activities measured using the Dual-glo Luciferase^®^ Reporter System (Promega, Madison, WI) and a 96-well plate luminometer (Dynex, Worthing, West Sussex, UK). RLUs were calculated as the quotient of Renilla / firefly RLU and normalized to mock.

### Protein immunoblots

Whole cell extracts of cells transfected with snoRNA-93 inhibitor or snoRNA-93 mimic or appropriate controls were used for western blot analysis of protein levels. The samples were fractionated by SDS-PAGE (15% resolving gel and 4% stacking gel) and transferred to a polyvinylidene difluoride (PVDF) membrane for 2 h. After incubation with 5% NFDM (nonfat dry milk) in TBST (20 mM Tris, pH 7.4, 150 mM NaCl, 2 mM KCl, 0.5% Tween-20) for 60 min, the PVDF membrane was incubated with antibodies against PIPOX (PA5-39333, Invitrogen Corp, Carlsbad, CA) (1:500 dilution) or beta-actin (MA5-15739-HRP, Thermo Fisher Scientific, Waltham, MA) (1:5000 dilution) at 4 °C overnight. Alternatively, when possible, membranes were instead simultaneously incubated with antibodies against PIPOX (1:500 dilution) and Vinculin (700062, Invitrogen) (1:5000 dilution) at 4 °C overnight. Membranes were washed with TBST three times for 5 min. Next, membranes were incubated with horseradish peroxidase-conjugated anti-rabbit antibody (32260, Thermo Fisher Scientific) diluted in 5% NFDM/TBST for 1 h at room temperature then washed with TBST 3 times for 5 min followed by a final wash in 0.2 M KPO_4_ (pH 8.4) for 5 min. Washed membranes were incubated in 2 mL of two chemiluminescent buffers ECL Bright (chemiluminescence) (AS14 ECL-100, Agrisera, Vännäs, Sweden) per manufacturer instructions for 1 min and then imaged on a LI-COR C-DiGit Chemiluminescent Blot Scanner.

### Small transcript northern blots

Total RNA from mimic transfected cells was isolated with Trizol^®^ (Life Sciences) per standard manufacture protocol. A 15% acrylamide/bis-acrylamide (29:1) gel containing 8 M urea (48% (w/v)) and 1× tris/borate/EDTA (TBE) buffer was prerun for 30 min at 100 V in a vertical mini- PROTEAN tank (Bio-Rad). Gels were flushed and loaded with 10 mg of total RNA in 2× TBE/Urea sample buffer (Bio-Rad), then run at 200 V until the bromophenol blue dye front reached the gel bottom. As a size reference, 1 µl of pooled, commercially synthesized biotin 5′ end-labeled DNA oligonucleotides (50, 20 and 10 bp each at 1 µM) was also loaded in 2× TBE/Urea sample buffer. After removal from the electrophoresis plates, gels were gently rinsed with water then washed in 0.5× TBE for 5 min on an orbital shaker. After electrophoresis, RNA was electro-transferred (Mini Trans-Blot Electrophoretic Transfer Cell apparatus, Bio-Rad) to Biodyne B Pre-cut Modified Nylon Membranes 0.45 µm (Thermo Scientific) for 2 h at 20 V in 0.5× TBE. After removal from the transfer stack, membranes were gently washed in 1× TBE for 15 min on an orbital shaker, then UV cross-linked at 1200 kJ (Stratalinker, Stratagene). Prehybridization was performed in North2South Hybridization Buffer (Thermo Scientific) at 42 °C for 30 min, after which 30 ng (per milliliter of hybridization buffer) of appropriate biotin 5’ end–labeled oligonucleotide (reverse complement to sdRNA-93) was added directly to the hybridization buffer as probe. Blots were hybridized overnight with gentle rotation at 42 °C. Hybridization buffer was removed the following day, and membranes washed and developed using the Thermo Scientific North2South Chemiluminescent Hybridization and Detection Kit per manufacturer instructions then imaged on a LI-COR C-DiGit Chemiluminescent Blot Scanner.

### Statistical analysis

#### QPCR

Data were analyzed by Fisher’s exact test depending on the Ct (cycle threshold) values included in qRT-PCR results. Differences were considered significant if the probability was less than 0.05. *Cell proliferation and invasion assays*: Five microscopic fields randomly chosen from each assay were counted individually, and the statistical significance between treatment and controls was determined by t-test (*P < 0.05). *Luciferase and western assays*: Data are presented as the average intensity ± standard deviation in three (western) or four (luciferase) independent experiments.

### Data availability

Total RNA sequencing was performed for MCF-7 and MDA-MB-231 to provide reads for all RNA transcripts. Similarly, small RNA-Seq was performed for MCF-7 and MDA-MB-231 to provide small RNAs ranging from 17 to 35 nucleotides in length. These data sets are available in the NCBI Sequence Read Archive repository under PRJNA390981 (www.ncbi.nlm.nih.gov/bioproject/390981). In addition to these, all other next-generation small RNA deep-sequencing libraries utilized are publicly available and were obtained from the NCBI Sequence Read Archive (SRA) (www.ncbi.nlm.nih.gov/sra/) as detailed under “Search for miRNAs derived from miRNA-like snoRNAs in SRA files” and “Quantifying sdRNA-93 expression in small RNA-Seq SRA files” above. All other relevant data (e.g., alignment files) are available from the authors upon request.

## Electronic supplementary material

Supplementary Figure 1

Supplementary Figure 2

Supplementary Figure 3

Supplementary Figure 4

Supplementary Table 1

Supplementary Table 2

Supplementary Table 3

Supplementary Table 4

Supplementary Table 5
