# Supplementary figures and images for: Human snoRNA-93 is processed into a microRNA-like RNA that promotes breast cancer cell invasion
Source: NPJ Breast Cancer. 2017 Jul 10;3:25. doi: 10.1038/s41523-017-0032-8 (PMC5503938; doi:10.1038/s41523-017-0032-8)

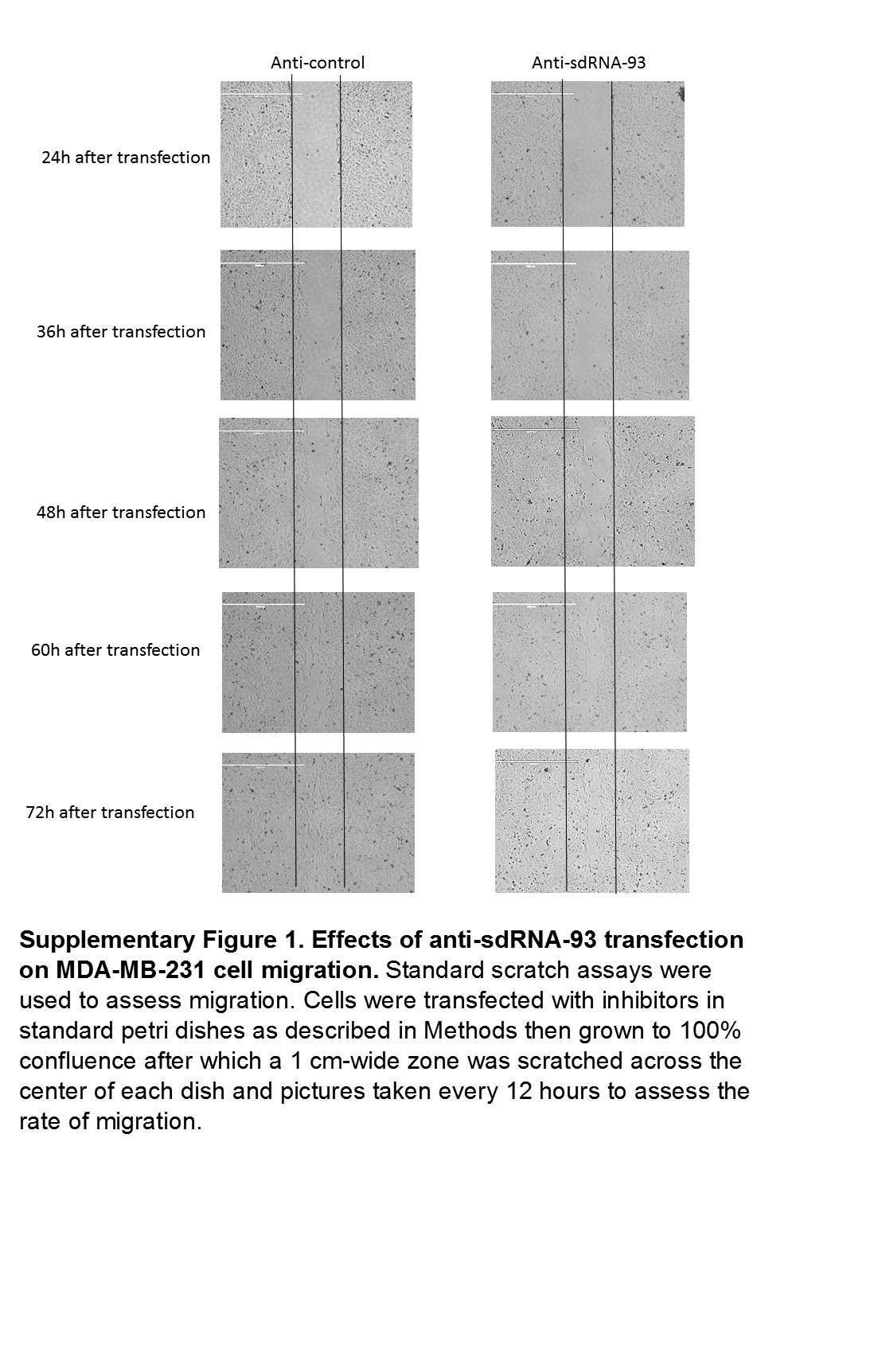

Supplement: Supplementary file 1 — Supplementary Figure 1 [file 41523_2017_32_MOESM1_ESM.tif]

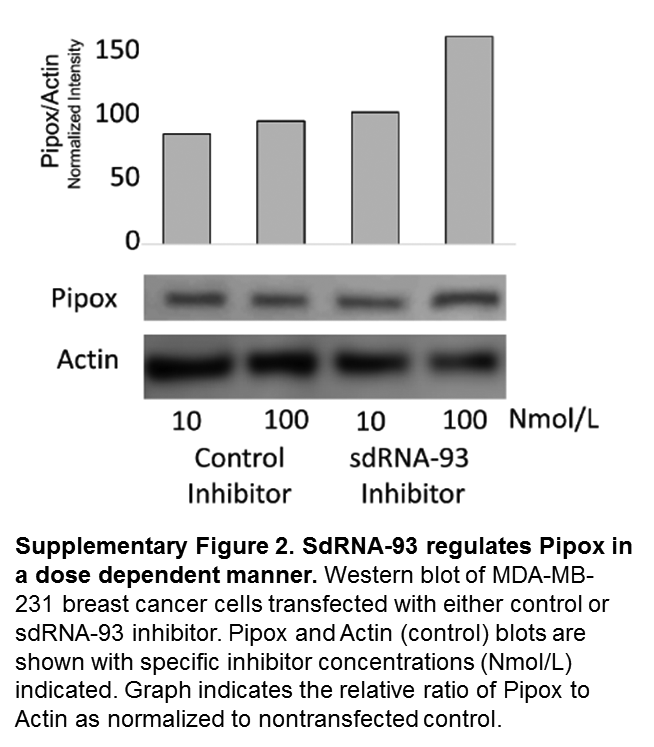

Supplement: Supplementary file 2 — Supplementary Figure 2 [file 41523_2017_32_MOESM2_ESM.tif]

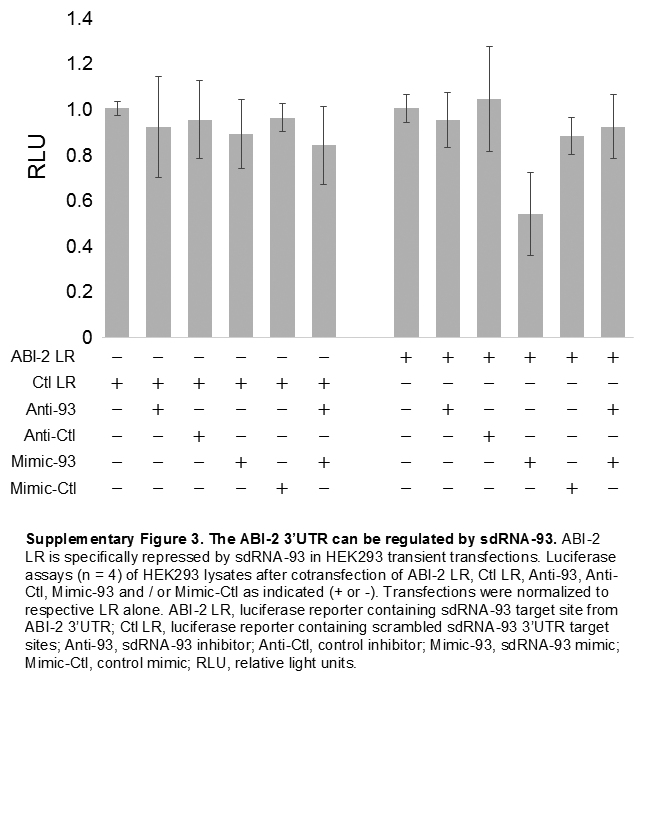

Supplement: Supplementary file 3 — Supplementary Figure 3 [file 41523_2017_32_MOESM3_ESM.tif]

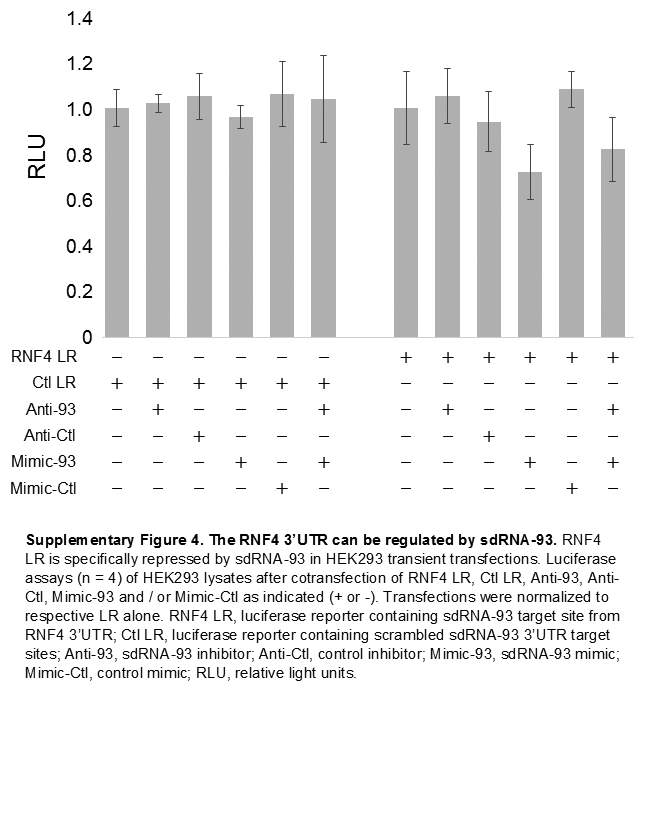

Supplement: Supplementary file 4 — Supplementary Figure 4 [file 41523_2017_32_MOESM4_ESM.tif]

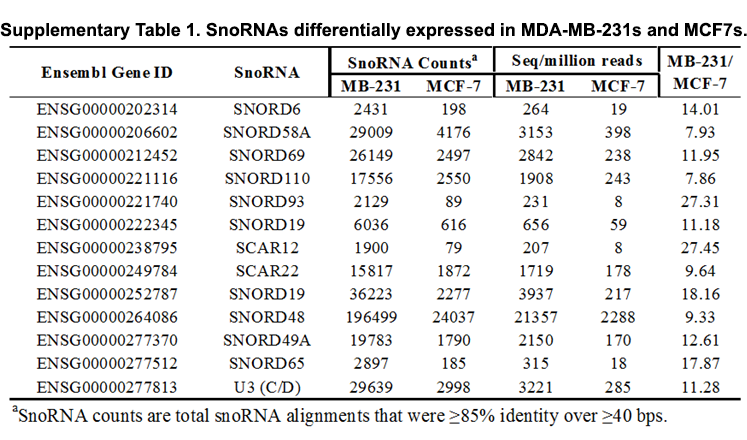

Supplement: Supplementary file 5 — Supplementary Table 1 [file 41523_2017_32_MOESM5_ESM.tif]

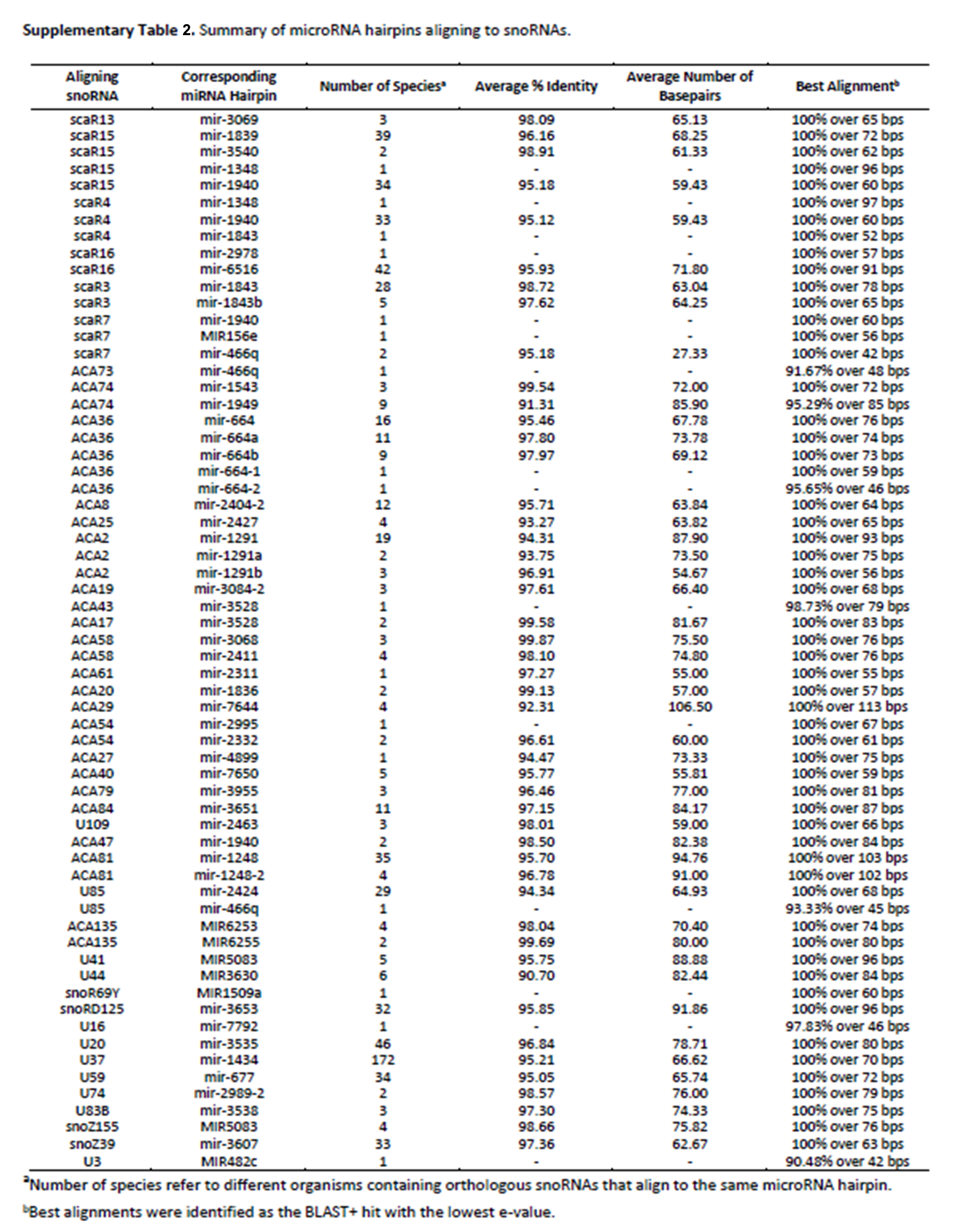

Supplement: Supplementary file 6 — Supplementary Table 2 [file 41523_2017_32_MOESM6_ESM.tif]

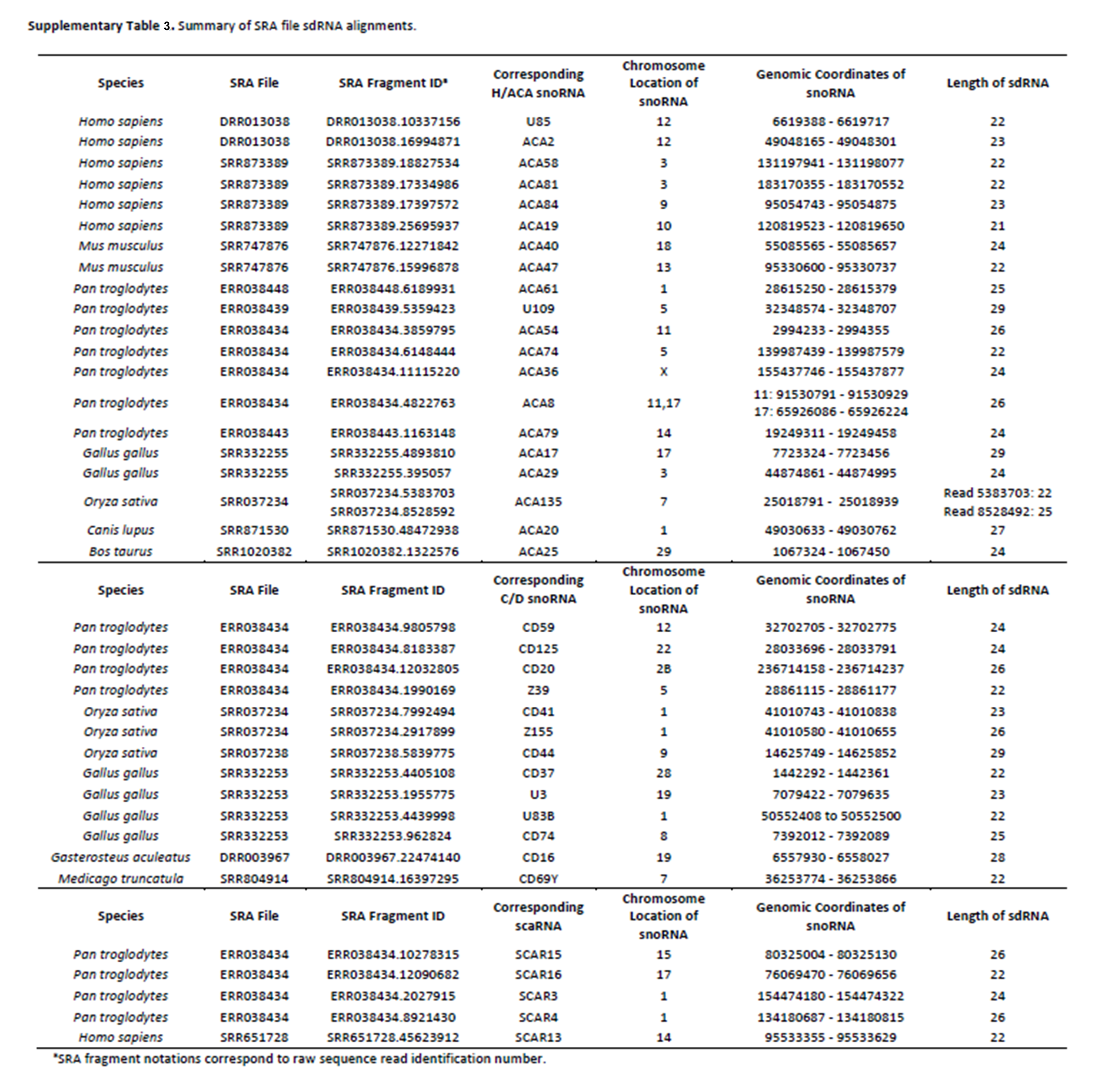

Supplement: Supplementary file 7 — Supplementary Table 3 [file 41523_2017_32_MOESM7_ESM.tif]

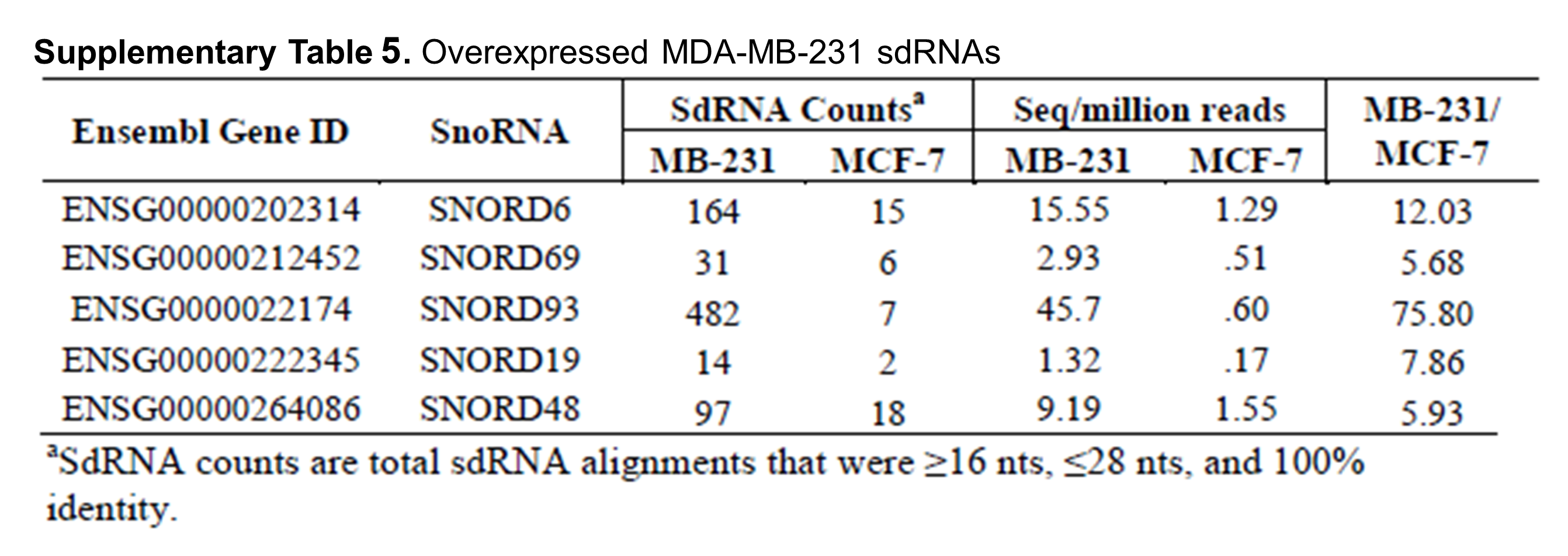

Supplement: Supplementary file 9 — Supplementary Table 5 [file 41523_2017_32_MOESM9_ESM.tif]
