# Supplementary material for: Human snoRNA-93 is processed into a microRNA-like RNA that promotes breast cancer cell invasion
Source: NPJ Breast Cancer. 2017 Jul 10;3:25. doi: 10.1038/s41523-017-0032-8 (PMC5503938; doi:10.1038/s41523-017-0032-8)
Supplement: Supplementary file 8 — Supplementary Table 4 [file 41523_2017_32_MOESM8_ESM.pdf]

Supplementary Table 4

A

```
Ch. 9             GAAGCAGGTGATGAAAGCAAGACAGGGACATCAGCACCCTCTTCTCTTTTGAGAGAGATGGGAATGATCGACTAAGTCTATAATGGGAATCTCTC
                  *****************|||||||||||||||||||||||||||||*************************************************
CD44              GAAGCAGGTGATGAAAGCAAGACAGGGACATCAGCACCCTCTTCTCTTTTGAGAGAGATGGGAATGATCGACTAAGTCTATAATGGGAATCTCTC
                                   |||||||||||||||||||||||||||||
SRR037238                          CAAGACAGGGACATCAGCACCCTCTTCTC
(Read: 5839775)
Ch. 28            GTTCTGCTGCAGTGCGTGATGATGGAAAATTTCTTCACTTTGACCTGACTTGTTTGATTGAAGAAATTTGAGTATCTGACGCGAGCTGAGCTCGT
                             ||||||||||||||||||||||************************************************
CD37                         GTGCGTGATGATGGAAAATTTCTTCACTTTGACCTGACTTGTTTGATTGAAGAAATTTGAGTATCTGACG
                             ||||||||||||||||||||||
SRR332253                    GTGCGTGATGATGGAAAATTTC
(Read: 4405108)
Ch. 19            AGGACTATACTTTCAGGGATCATTTCTGTAGTTGGTAACTAGAGGAGTGTGATCGAAAGTGTCCGGCGTCACAAACCACGAGGAGGAGCAGCGTG
                  **|||||||||||||||||||||||**********************************************************************
U3                AGGACTATACTTTCAGGGATCATTTCTGTAGTTGGTAACTAGAGGAGTGTGATCGAAAGTGTCCGGCGTCACAAACCACGAGGAGGAGCAGCGTG
                    |||||||||||||||||||||||
SRR332253           GACTATACTTTCAGGGATCATTT
(Read: 1955775)
Ch. 1             CGCAGTTCGGTGATGAAACCATGGAATAAGCGCTGGGCACAGCGCCTGACATCCGTGAGGAGTCGTTCCATGCTGCCTTCCTTCTGAGAACAACA
                   *********************************************************************||||||||||||||||||||||**
U83B               GCAGTTCGGTGATGAAACCATGGAATAAGCGCTGGGCACAGCGCCTGACATCCGTGAGGAGTCGTTCCATGCTGCCTTCCTTCTGAGAACAAC
                                                                                        ||||||||||||||||||||||
SRR332253                                                                               TGCTGCCTTCCTTCTGAGAACA
(Read: 4439998)
Ch. 8             CATTCCATGTGCCCGCACATGATGAAAACTATGTTGGTAGGGACATCTGAGAGGCTGATGAGTACCAACATATCTGAGCTGGGCATGTAGCAGGG
                          ****************************|||||||||||||||||||||||||**************************
CD74                      GTGCCCGCACATGATGAAAACTATGTTGGTAGGGACATCTGAGAGGCTGATGAGTACCAACATATCTGAGCTGGGCAT
                                                      |||||||||||||||||||||||||
SRR332253                                             GTAGGGACATCTGAGAGGCTGATGA
(Read: 962824)
Ch. 19            TGATGGTGTAATTTGCGACTTACTCTGTGATCAGTGACAGATGCCTACTGTCATATGCTGGCACAGATGGCTGACGAACAATGAGTCTGAGCAAG
                  *****************************************************************||||||||||||||||||||||||||||
CD16              TGATGGTGTAATTTGCGACTTACTCTGTGATCAGTGACAGATGCCTACTGTCATATGCTGGCACAGATGGCTGACGAACAATGAGTCTGAGCA
                                                                                   ||||||||||||||||||||||||||||*
DRR003967                                                                          GATGGCTGACGAACAATGAGTCTGAGCAA
(Read: 22474140)
Ch. 7             TTTAGCTATGATGACAATGTGTATCCCAGCTCATTATGAGGCCTTTTTGTAAGGTCTGGGAATTTAACCTTCCACACATTTATCTGAGCTTTTCT
                  **||||||||||||||||||||||**********************************************************************
CD69Y             TTTAGCTATGATGACAATGTGTATCCCAGCTCATTATGAGGCCTTTTTGTAAGGTCTGGGAATTTAACCTTCCACACATTTATCTGAGCTTTTC
                    ||||||||||||||||||||||
SRR804914           TAGCTATGATGACAATGTGTAT
(Read: 16397295)
```

```
Ch. 12            ATTGACATCTATTCCTCACTGATGAGTACGTTCTGACTTTCGTTCTTCTGAGTTTGCTGAAGCCAGATGCAATTTCTGAGAAGGAATAGGATGGA
                               *||||||||||||||||||||||||**********************************************
CD59                           CCTCACTGATGAGTACGTTCTGACTTTCGTTCTTCTGAGTTTGCTGAAGCCAGATGCAATTTCTGAGAAGG
                                ||||||||||||||||||||||||
ERR038434                       CTCACTGATGAGTACGTTCTGACT
(Read: 9805798)
Ch. 22            ACCCCTGGCAGCCCCTCCTGATGATTCTTCTTCCTGAGCACGCTCATGATGAGCAAACTGAGCCTCTAAGAAGTTGACTGAAGGGGCTGCTTCCC
                  *******************************************||||||||||||||||||||||||****************************
CD125             ACCCCTGGCAGCCCCTCCTGATGATTCTTCTTCCTGAGCACGCTCATGATGAGCAAACTGAGCCTCTAAGAAGTTGACTGAAGGGGCTGCTTCCC
                                                             ||||||||||||||||||||||||
ERR038434                                                    TCATGATGAGCAAACTGAGCCTCT
(Read: 8183387)
Ch. 2B            TGGATATGATGACTGATTACCTGAGAAATAATTGATGAAATCTCAAGAAAATTCCTCTAGATAGTCAAGTTCTGATCCAGCTGTGTCAACTCAGA
                  ||||||||||||||||||||||||||******************************************************
CD20              TGGATATGATGACTGATTACCTGAGAAATAATTGATGAAATCTCAAGAAAATTCCTCTAGATAGTCAAGTTCTGATCCAG
                  ||||||||||||||||||||||||||
ERR038434         TGGATATGATGACTGATTACCTGAGA
(Read: 12032805)
Ch. 5             TGCGGAAAGGTTGCGGTGCATGTGATGAAGCAAATCAGTATGAATGAATTCATGATACTGTAAACGCTTTCTGATGTACTACTCAAACTGATAGA
                                 ********************************||||||||||||||||||||||*********
Z39                              GTGCATGTGATGAAGCAAATCAGTATGAATGAATTCATGATACTGTAAACGCTTTCTGATGTA
                                                                 ||||||||||||||||||||||
ERR038434                                                        ATTCATGATACTGTAAACGCTT
(Read: 1990169)
Ch. 1             TTGGAGGCTGTGATGACCAAAAAATACCTACCTATTTTCTGAGGGATTCCAGTCCTTCTGATTGATAGAAACCAATGGATCCTTCTGAGCCTGCC
                  ****|||||||||||||||||||||||********************************************************************
CD41              TTGGAGGCTGTGATGACCAAAAAATACCTACCTATTTTCTGAGGGATTCCAGTCCTTCTGATTGATAGAAACCAATGGATCCTTCTGAGCCTGCC
                      |||||||||||||||||||||||
SRR037234             AGGCTGTGATGACCAAAAAATAC
(Read: 7992494)
Ch. 1             AGTTGATGGGATGATAACCTTTCACAGAAACTGTATGTGTTGACCCAAAATTCAGACTACAATTATCTGATCAACTCAGACATCTTGCTAAAGGG
                  **||||||||||||||||||||||||||************************************************
Z155              AGTTGATGGGATGATAACCTTTCACAGAAACTGTATGTGTTGACCCAAAATTCAGACTACAATTATCTGATCAACT
                    ||||||||||||||||||||||||||
SRR037234           TTGATGGGATGATAACCTTTCACAGA
(Read: 2917899)
```

B

```
Ch. 5               TGTCTGCTTCTTATTCCTTGGGAGCAGGAATATGTTCATAACATGCTACATTAACAAAGGAGTTCTCAGGGCTGCCAACCTTCTAGTAAAGGTTG
                    *******************************************************|||||||||||||||||||||||||||||***********
U109                TGTCTGCTTCTTATTCCTTGGGAGCAGGAATATGTTCATAACATGCTACATTAACAAAGGAGTTCTCAGGGCTGCCAACCTTCTAGTAAAGGTTG
                                                                           |||||||||||||||||||||||||||||
ERR038439                                                                  AAAGGAGTTCTCAGGGCTGCCAACCTTCT
(Read: 5359423)
Ch. 14              TGATGGCTGTTCCTCTCACTGCTTGAAGCCTTAGGCAGTGGGATTTTGATCCATCATATATCAAAAATGGCTTATCTTCACTCAGGGCACCAT
                    ||||||||||||||||||||||||*********************************************************************
ACA79               TGATGGCTGTTCCTCTCACTGCTTGAAGCCTTAGGCAGTGGGATTTTGATCCATCATATATCAAAAATGGCTTATCTTCACTCAGGGCACCAT
                    ||||||||||||||||||||||||
ERR038443           TGATGGCTGTTCCTCTCACTGCTT
(Read: 1163148)
Ch. 1               GGTCTTGGTGGTCGTAAAAGGAGGAAAAGTAATAGTGAAGCTGGCCTAAATGTTGTAATCTGGTATATGGCATGTGGGCTAGTTTCAGACAGGTT
                    *********************************************************************|||||||||||||||||||||||||
ACA61               GGTCTTGGTGGTCGTAAAAGGAGGAAAAGTAATAGTGAAGCTGGCCTAAATGTTGTAATCTGGTATATGGCATGTGGGCTAGTTTCAGACAGGT
                                                                                         |||||||||||||||||||||||||*
ERR038448                                                                                GCATGTGGGCTAGTTTCAGACAGGTT
(Read: 17334986)
Ch. 7               CATGCAGGACTAGTATAAATGGAGCTGCACATACAATTTCATTGTACATGCTGCTTAAACTTTCCTGCTAAACCAGTTTTGGGAAAAAATGGGCAG
                    ||||||||||||||||||||||**************************************************************************
ACA135              CATGCAGGACTAGTATAAATGGAGCTGCACATACAATTTCATTGTACATGCTGCTTAAACTTTCCTGCTAAACCAGTTTTGGGAAAAAATGGGCAG
                    ||||||||||||||||||||||
SRR037234           CATGCAGGACTAGTATAAATGG
(Read: 5383703)
Ch. 7               TTAAACTTTCCTGCTAAACCAGTTTTGGGAAAAAATGGGCAGTTGAGTTCGGCCAATTTATCTATGGTTTCCTCAACAATCATTTTTCCCACATGT
                    ***********************************************************************|||||||||||||||||||||||||
ACA135*             TTAAACTTTCCTGCTAAACCAGTTTTGGGAAAAAATGGGCAGTTGAGTTCGGCCAATTTATCTATGGTTTCCTCAACAATCATTTTTCCCACATGT
                    ***********************************************************************|||||||||||||||||||||||||
SRR037234                                                                                  CTCAACAATCATTTTTCCCACATGT
(Read: 8528592)
Ch. 17              ATGTTTGTGTCATTAGGTGGCAGAAAGGAAAAGGCTGTGTCTTTGCTAATGCTCTGAAACCGGTGAGCACTCAGGAATGACTAGCAACCTGACAA
                    ********************************************************|||||||||||||||||||||||||||||**********
ACA17               ATGTTTGTGTCATTAGGTGGCAGAAAGGAAAAGGCTGTGTCTTTGCTAATGCTCTGAAACCGGTGAGCACTCAGGAATGACTAGCAACCTGACAA
                                                                            |||||||||||||||||||||||||||||
SRR332255                                                                   AAACCGGTGAGCACTCAGGAATGACTAGC
(Read: 4893810)
Ch. 3               TCCCATTATACTACCACTTTTTCAATCTCCAGAGGAAGTGGCTATGCTTGTGGGATAGAGGAAAATGTTGCCACGGTCTGAAGGAAAAGGTTGAT
                    *******************************************************************||||||||||||||||||||||||****
ACA29               TCCCATTATACTACCACTTTTTCAATCTCCAGAGGAAGTGGCTATGCTTGTGGGATAGAGGAAAATGTTGCCACGGTCTGAAGGAAAAGGTTGAT
                                                                                       ||||||||||||||||||||||||
SRR332255                                                                              TTGCCACGGTCTGAAGGAAAAGGT
(Read: 395057)
```

```
Ch. 18              TGAGCCAGTTGGACAGGTACAAAAATAAATCCTCTTTTGCAACCCAGAACTCATTGCTCAGTATGAGTTTTGATACATACAAGAAGGGATATG
                    *******************************||||||||||||||||||||||||**************************************
ACA40               TGAGCCAGTTGGACAGGTACAAAAATAAATCCTCTTTTGCAACCCAGAACTCATTGCTCAGTATGAGTTTTGATACATACAAGAAGGGATATG
                                                   ||||||||||||||||||||||||
SRR747876                                          CTCTTTTGCAACCCAGAACTCATT
(Read: 12271842)
Ch. 13              GAAGGTGGAGCAGTTCTGTACCGTGCTGCCTTCCATTGGTTAAGACCTCCAACAGTAAAGGGCTGGCCGGCCAAAGAATGTCCTCTCTATCACCG
                    **************************************************************||||||||||||||||||||||***********
ACA47               GAAGGTGGAGCAGTTCTGTACCGTGCTGCCTTCCATTGGTTAAGACCTCCAACAGTAAAGGGCTGGCCGGCCAAAGAATGTCCTCTCTATCACCG
                                                                                  ||||||||||||||||||||||
SRR747876                                                                         CTGGCCGGCCAAAGAATGTCCT
(Read: 15996878)
Ch. 12              AGTAGTGTTATTTCTTATGTGCTATACAAATAATTGAAGGCTAATTAGCAGTATAACTATAAATAGTAATGCTGCCAGTCTCCTTCAGACAAAAA
                    *********************************************************************|||||||||||||||||||||||***
ACA19               AGTAGTGTTATTTCTTATGTGCTATACAAATAATTGAAGGCTAATTAGCAGTATAACTATAAATAGTAATGCTGCCAGTCTCCTTCAGACAAAAA
                                                                                         |||||||||||||||||||||||
SRR873389                                                                                TGCTGCCAGTCTCCTTCAGACAA
(Read: 25695937)
Ch. 3               CAGGGGGGACAGTGTATGCAAGAATAATTTGGAGTTCCTGCCAGCTCTAACCAGCTTCATCAGTGGCTGGATAAATTGCAGGACTCTAAACATTT
                    *************************************************************************||||||||||||||||||||||
ACA58               CAGGGGGGACAGTGTATGCAAGAATAATTTGGAGTTCCTGCCAGCTCTAACCAGCTTCATCAGTGGCTGGATAAATTGCAGGACTCTAAACATTT
                                                                                             ||||||||||||||||||||||
SRR873389                                                                                    AATTGCAGGACTCTAAACATTT
(Read: 18827534)
Ch. 1               TCCCATTGATTTGCTGCTCTAGTCTTTCAGTGATAGGAGCAGTTATACGCATGGGAGAGAACAATGTTAGGCCATGGTAGATAGAGATGGAGTAC
                    *******************************************************************|||||||||||||||||||||||||||*
ACA20               TCCCATTGATTTGCTGCTCTAGTCTTTCAGTGATAGGAGCAGTTATACGCATGGGAGAGAACAATGTTAGGCCATGGTAGATAGAGATGGAGTAC
                                                                                       |||||||||||||||||||||||||||
SRR871530                                                                              TAGGCCATGGTAGATAGAGATGGAGTA
(Read: 48472938)
Ch. 29              AGGTCATTTCAAAGAGGGCTGATGGGACCAAATCCTGGAGCCCTTAACGCTGTGACCAAAGACTGAATTCTCCATTGGATATAATAGTCACTTGA
                    *******************************************************************||||||||||||||||||||||||****
ACA25               AGGTCATTTCAAAGAGGGCTGATGGGACCAAATCCTGGAGCCCTTAACGCTGTGACCAAAGACTGAATTCTCCATTGGATATAATAGTCACTTGA
                                                                                       ||||||||||||||||||||||||
SRR1020382                                                                             TTCTCCATTGGATATAATAGTCAC
(Read: 1322576)
Ch. 12              GTGGCCCTGACTGAAGACCAGCAGTTGTACTGTGGCTGTTGGTTTCAAGCAGAGGCCTAAAGGACTGTCTTCCTGTGGTCTGTTGGCTGTTCTGG
                    |||||||||||||||||||||||************************************************************************
ACA2                GTGGCCCTGACTGAAGACCAGCAGTTGTACTGTGGCTGTTGGTTTCAAGCAGAGGCCTAAAGGACTGTCTTCCTGTGGTCTGTTGGCTGTTCTGG
                    |||||||||||||||||||||||
DRR013038           GTGGCCCTGACTGAAGACCAGCA
(Read: 16994871)
```

```
Ch. 9         TGCCCTGTGGTTGCTGGATGCTGTTGTGCATGGACAGCTCTCCAGTGGATTCGATGGGCCATAGCAATCCTGTGATTTATGCATGGAGGCTGCTTC
               |||||||||||||||||||||||************************************************************************
ACA84          GCCCTGTGGTTGCTGGATGCTGTTGTGCATGGACAGCTCTCCAGTGGATTCGATGGGCCATAGCAATCCTGTGATTTATGCATGGAGGCTGCTTC
              *|||||||||||||||||||||||
SRR873389     TGCCCTGTGGTTGCTGGATGCTGT
(Read: 17397572)
Ch. 3         CCAGATAGAAAAACTGAGTTGAACCTTTTTTTTTTCTTTTTTTTAGATGGAGTCTCACTCTGTCACCCAGGCTGGAGTGTAGTGGCGCGATCTCA
              *********************************************************||||||||||||||||||||||****************
ACA81         CCAGATAGAAAAACTGAGTTGAACCTTTTTTTTTTCTTTTTTTTAGATGGAGTCTCACTCTGTCACCCAGGCTGGAGTGTAGTGGCGCGATCTCA
                                                                       ||||||||||||||||||||||
SRR873389                                                              CTCTGTCACCCAGGCTGGAGTG
(Read: 17334986)
Ch. 12        GATGATATCAAGGCTGTTGTGATTCAGTTGGTTTGGCTAAGCCCAGGGACCTTTGGCCTGTTAAAGGTCTGTAATCTTGGTGGGCGATACAGAGT
              ***************************************************************||||||||||||||||||||||**********
U85           GATGATATCAAGGCTGTTGTGATTCAGTTGGTTTGGCTAAGCCCAGGGACCTTTGGCCTGTTAAAGGTCTGTAATCTTGGTGGGCGATACAGAGT
              ***************************************************************||||||||||||||||||||||**********
DRR013038                                                                    AAGGTCTGTAATCTTGGTGGGC
(Read: 10337156)
Ch. 5         ACTGCTGGGAGAACAGCAGCCAATAGCTGGTTGGCATTCTGGCCCTGGTTCATGCCAACTCTTGTGTTGACTACCCCAGGATGCCAGCATAGTTG
              *********************************************************************||||||||||||||||||||||
ACA74         ACTGCTGGGAGAACAGCAGCCAATAGCTGGTTGGCATTCTGGCCCTGGTTCATGCCAACTCTTGTGTTGACTACCCCAGGATGCCAGCATA
                                                                                   ||||||||||||||||||||||**
ERR038434                                                                          ACTACCCCAGGATGCCAGCATAGT
(Read: 6148444)
Ch. X         TTCCAAAGTGTTGAGTTCAGTCCAGGGCAGCTTCCCTGTTCTGTTAATTAAACTTTGGGACATTAAAATGGGCTAAGGGAGATGATTGGGTAGAA
              *********************************************************************|||||||||||||||||||||||***
ACA36         TTCCAAAGTGTTGAGTTCAGTCCAGGGCAGCTTCCCTGTTCTGTTAATTAAACTTTGGGACATTAAAATGGGCTAAGGGAGATGATTGGGTAGAA
                                                                                   |||||||||||||||||||||||
ERR038434                                                                          GGGCTAAGGGAGATGATTGGGTA
(Read: 11115220)
Ch. 11,17     TGCACTGCATGGCATCTGCACCCAGCAGTTTATTCTTGCTAAGGTGTTCAAAGGTCAGTGCTATAGAAATTCAGTATCTGGCATCATTGGTTTTC
              ********************************************************||||||||||||||||||||||||||*************
ACA8          TGCACTGCATGGCATCTGCACCCAGCAGTTTATTCTTGCTAAGGTGTTCAAAGGTCAGTGCTATAGAAATTCAGTATCTGGCATCATTGGTTTTC
                                                                      ||||||||||||||||||||||||||
ERR038434                                                             AGTGCTATAGAAATTCAGTATCTGGC
(Read: 4822763)
```

C

```
Ch. 15          CTGGAGACTAAGAAAATAGAGTCCTTGAAATCAAGCTGACTCTGCTTTTAGCCTCCTAAATGAAAAGGTAGATAGAACAGGTCTTGTTTGCAAAA
                *****************************************************************||||||||||||||||||||||||||****
SCAR15          CTGGAGACTAAGAAAATAGAGTCCTTGAAATCAAGCTGACTCTGCTTTTAGCCTCCTAAATGAAAAGGTAGATAGAACAGGTCTTGTTTGCAAAA
                                                                                 ||||||||||||||||||||||||||
ERR038434                                                                        AGGTAGATAGAACAGGTCTTGTTTGC
(Read:10278315)
Ch. 17          CCCGGGAGAAGTCTTTCCTGGGTTTTGAATTTGCAGTAACAGGTGTGAGCATTCTAGCAGCAGTTTGATGATCATGTATGATACTGCAAACAGGA
                **********************************************************************||||||||||||||||||||||***
SCAR16          CCCGGGAGAAGTCTTTCCTGGGTTTTGAATTTGCAGTAACAGGTGTGAGCATTCTAGCAGCAGTTTGATGATCATGTATGATACTGCAAACAGGA
                                                                                      ||||||||||||||||||||||
ERR038434                                                                             ATCATGTATGATACTGCAAACA
(Read:12090682)
Ch. 1           TGCTGGAACCAGCCATGTGGACAAATGAAAAAAAGCCTTTTGTCTACAGATTGCAGCGATCCTACATAAACATATGGAGGTCTCTGTCTGGCTTA
                ***********************************************************************||||||||||||||||||||||||
SCAR3           TGCTGGAACCAGCCATGTGGACAAATGAAAAAAAGCCTTTTGTCTACAGATTGCAGCGATCCTACATAAACATATGGAGGTCTCTGTCTGGCTTA
                                                                                       ||||||||||||||||||||||||
ERR038434                                                                              ATATGGAGGTCTCTGTCTGGCTTA
(Read: 2027915)
Ch. 1           ACTGGAGGACTAAGAAGGCTGAGTCTGATGAAGTAAGACTTTGCTGATACATTCCTCCTAGAAAAAAGGGTTGGAGAGAGCAGCCTTCACTGAAG
                *||||||||||||||||||||||||||********************************************************************
SCAR4           ACTGGAGGACTAAGAAGGCTGAGTCTGATGAAGTAAGACTTTGCTGATACATTCCTCCTAGAAAAAAGGGTTGGAGAGAGCAGCCTTCACTGAAG
                 ||||||||||||||||||||||||||
ERR038434        CTGGAGGACTAAGAAGGCTGAGTCTG
(Read: 8921430)
Ch. 14          AATCTGTAGTCTTGGAGCCGCACAGGGTTGGTGGTACCCTCGAGCACACCAGACTTGCAGAAAAAGCATACTCCAGAGGAAGCTGAGGCATGCCT
                *******************************************************************||||||||||||||||||||||******
SCAR13          AATCTGTAGTCTTGGAGCCGCACAGGGTTGGTGGTACCCTCGAGCACACCAGACTTGCAGAAAAAGCATACTCCAGAGGAAGCTGAGGCATGCCT
                                                                                   ||||||||||||||||||||||
SRR651728                                                                          ATACTCCAGAGGAAGCTGAGGC
(Read: 45623912)
```

Supplementary Table 4. Tripartite alignments of small RNA generating snoRNAs.

Alignments between the genome (top), snoRNA (middle), and small RNA reads mined from various SRA files (bottom) are illustrated. All sequences are in the 5' to 3' direction. The asterisk indicates base identity with the genome. Vertical lines demonstrate three aligning sequences. (**A**) C/D snoRNA sequences correspond to the following nucleotide positions: CD44 (1-95), CD37 (1-70), U3 (1-95), U83B (1-93), CD74 (1-78), CD16 (6-98), CD69Y (1-95), CD59 (1-71), CD125 (1-95), CD20 (1-80), Z39 (1-63), CD41 (1-95), Z155 (1-76). (**B**) H/ACA snoRNA sequences correspond to the following nucleotide positions: U109 (14-108), ACA79 (1-93), ACA61 (37-130), ACA135 (1-95), ACA135* (55-149), ACA17 (38-132), ACA29 (1-95), ACA40 (1-93), ACA47 (12-106), ACA19 (34-129), ACA58 (43-137), ACA20 (3-97), ACA25 (1-95), ACA2 (1-95), ACA84 (1-95), ACA81 (103-198), U85 (8-102), ACA74 (51-141), ACA36 (1-95), ACA8 (1-95). (**C**) ScaRNA sequences correspond to the following nucleotide positions: SCAR15 (1-95), SCAR16 (93-187), SCAR3 (10-104), SCAR4 (1-95), SCAR13(1-95).
